# A Computational Multiscale Framework for Bone Remodeling: Coupling Apparent Density Evolution and Microscale Shape Optimization

**DOI:** 10.1002/cnm.70097

**Published:** 2025-10-19

**Authors:** Balavignesh Vemparala, Mingshi Ji, Prasath Mageswaran, Gregory G. Knapik, Khaled Dibs, Dukagjin M. Blakaj, Eric C. Bourekas, Ehud Mendel, William S. Marras, Soheil Soghrati

**Affiliations:** ^1^ Department of Mechanical and Aerospace Engineering The Ohio State University Columbus Ohio USA; ^2^ Department of Integrated Systems Engineering The Ohio State University Columbus Ohio USA; ^3^ Department of Radiation Oncology The Ohio State University Columbus Ohio USA; ^4^ Department of Neurological Surgery The Ohio State University Columbus Ohio USA; ^5^ Department of Radiology The Ohio State University Columbus Ohio USA; ^6^ Department of Neurosurgery Yale University School of Medicine New Haven Connecticut USA; ^7^ Department of Materials Science and Engineering The Ohio State University Columbus Ohio USA

**Keywords:** bone remodeling, finite element method, mechanobiological, radiation therapy, vertebral compression fracture

## Abstract

Bone remodeling models are typically phenomenological or mechano‐biological but often lack mechanisms to incorporate patient‐specific data, limiting clinical use. We present a patient‐specific multiscale framework that couples finite element (FE)‐based shape optimization at the microscale with a mechano‐biological model at the macroscale. The model predicts % bone mineral density (BMD) changes at the macroscale, which in turn drive microscale trabecular adaptation via % bone volume fraction (BV/TV) changes. Micro‐QCT imaging data are used to train a DCGAN‐based ReconGAN for virtual reconstruction of trabecular microstructures, from which FE models are generated. Apparent BMD changes predicted by the macroscale model guide the microscale shape optimization to simulate adaptation. The framework reproduces BMD losses of 9.8% (trabecular) and 4.9% (whole vertebra) over a 215‐day spaceflight scenario, consistent with results from prolonged bed rest and controlled experimental datasets. In vertebral compression fracture simulations, it captures trabecular bone degeneration by reducing peak load from 3.532 to 3.280 kN and energy absorption from 0.243 to 0.218 J, and recovery restores close agreement to the original microstructure. These results demonstrate a path toward patient‐specific simulation of bone remodeling and its mechanical consequences, with strong potential for treatment planning and assessment of skeletal interventions.

## Graphical abstract

This study introduces a multiscale patient‐specific framework that couples mechano‐biological bone remodeling at the macroscale with microscale shape optimization. The framework is validated through literature case studies and demonstrates how remodeling influences vertebral fracture behavior.

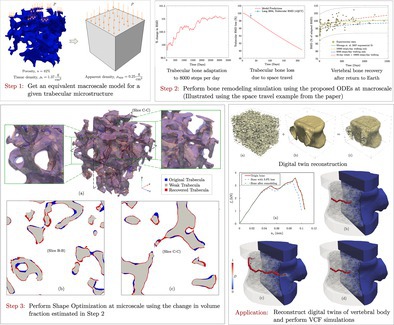

## 1 Introduction

Human bone is a composite structure, composed of inorganic mineral crystals analogous to Hydroxyapatite (HA), an extracellular organic matrix, cells, lipids, and water [1]. The cells that produce, nurture, and remodel the mineralized organic matrix, primarily composed of type I collagen, also respond to external mechanical loading and other signals, which determine the bone's mechanical properties and morphology [1].

Bone remodeling can be both directed and stochastic [2]. Directed remodeling usually occurs when the bone is subject to fatigue damage loading, where the osteocytes act as *sensors* (sensing the mechanical load) that trigger the recruitment of osteoclast precursor cells to the bone surface [2, 3]. Random remodeling is believed to occur during mineral homeostasis to ensure all parts of the bone are remodeled periodically [3, 4].

The bone multicellular unit (BMU), which is an approximately 2 mm long and 0.2 mm wide cylindrical structure, burrows through the bone at a rate of 20–40 μm/day [2, 3] during the remodeling process. The lifespan of a BMU is 6–12 months, starting with about nine osteoclasts resorbing bone at the forward end and ending with 2000 osteoblasts forming bone within the cavity [2]. The succession of events at a single cross‐section, starting from the beginning of resorption to the end of formation, represents a single cycle of remodeling [2]. Understanding and predicting the evolution of osteocytes, osteoclasts, and osteoblasts through modeling can provide insights into the dynamics of bone remodeling and lead to the development of better clinical treatment algorithms.

Several researchers have developed finite element (FE)‐based models to study bone remodeling. Wolff observed the self‐organizational nature of bone, that is, its ability to transform morphology in response to applied loads [5]. Many FE models rely on phenomenological remodeling laws based on this observation [6, 7, 8, 9, 10, 11]. However, the issue with an element‐based implementation of such phenomenological models is a numerical instability also referred to as the *checkerboarding* of the apparent density [12], occurred in the vicinity of the applied load. Jacobs et al. proposed the use of a node‐based implementation to address this issue [12]. Alternatively, Mullender et al. showed that checkerboarding is a mesh‐dependent instability and can be alleviated by separating the sensor density and range of action from the mesh [13]. This was also the first model that incorporated the effect of osteocytes in a phenomenological model, which was the first step towards a new class of models, that is, mechanobiological models. Recently, Martinez‐Reina et al. [14] showed that using linear models leads to non‐uniqueness of the converged apparent density field for different initializations of this field. They proposed the use of saturation‐type models to ensure the uniqueness of the solution. There are also studies focused on the best candidate for the mechanical stimulus function. The recent work by Zhang and Luo [15] is worth mentioning on this topic, in which they concluded that the strain energy density (SED) can best reproduce the femoral BMD distribution in a phenomenological model.

Concerning mechanobiological models, Bonfoh et al. [16] used the Komarova cell dynamics formulation [4] and proposed a framework coupling the evaluation of paracrine factors to the mechanical stimulus, chosen as SED. More recently, Rapisarda et al. [17] proposed a mechanobiological framework relying on a coupled system of differential equations to predict the evolution of osteocytes, osteoclasts, and osteoblasts for cell dynamics, coupled with a mechanical model. The novelty of this model compared to the Komarova model is the consideration of several signal transduction pathways, in which osteocytes influence the remodeling process [17]. Building on these approaches, Mertiya et al. [18] developed a computational model to assess the osteogenic potential of physical exercises based on mechanobiological environments, demonstrating the relationship between loading and cortical bone remodeling. Similarly, Peyroteo et al. [19] introduced a mechanobiological model that adapts bone remodeling as a function of applied loads, emphasizing the interplay between mechanical stimuli and biological responses. In a related study, Peyroteo et al. [20] proposed a meshless modeling framework using the Natural Neighbor Radial Point Interpolation Method (NNRPIM) to simulate bone remodeling under mechanical loads, achieving localized adaptation and optimized trabecular structures.

Additionally, Sansalone et al. [21] introduced a macroscopic model for bone remodeling using generalized continuum mechanics, which incorporates both the orientation of bone microstructure and mineral turnover within a thermodynamically consistent framework. Ramtani et al. [22] proposed an extended Komarova‐based model to study the interaction between tumors and bone remodeling, capturing the influence of tumor‐induced paracrine signaling on osteoclast and osteoblast dynamics. These recent contributions highlight the growing effort to integrate multiphysics and multiscale effects in bone adaptation models.

Apart from such deterministic models, some studies have considered the remodeling process as a mixture of stochastic and directed events [23, 24]. There are also several studies attempting to replicate bone remodeling as an optimization process [25, 26, 27, 28, 29, 30, 31].

In this work, several knowledge gaps in existing approaches to predicting the bone remodeling process are addressed. One of the limitations of the existing models in the literature is the lack of thorough validation studies, demonstrating their effectiveness in real‐life scenarios. Moreover, most of these models do not have a mechanism to incorporate patient‐specific parameters, which are crucial for application in clinical settings. The current study builds on the model proposed by Rapisarda et al. [17], and proposes some modifications to improve the accuracy. Further, a rigorous preliminary validation study and sensitivity analysis on the proposed model have been performed based on data available in the literature, including bone loss and recovery in NASA astronauts, bed rest studies, and two different controlled experiments on post‐menopausal women.

The remainder of this manuscript is structured as follows: Section 2 outlines the methodology, including the imaging procedure and microstructure reconstruction. Section 3 describes the model formulation by Rapisarda et al. and the modifications introduced for bone mineral density (BMD) estimation at the macroscale, along with the surface remodeling procedure implemented at the microscale. Section 4 presents the simulations conducted for model validation and discusses the results. Section 5 addresses the limitations of the proposed model. Final concluding remarks are provided in Section 6.

## 2 Material and Methods

### 2.1 Micro‐QCT Imaging and Trabecular Bone Reconstruction

**FIGURE 1:**
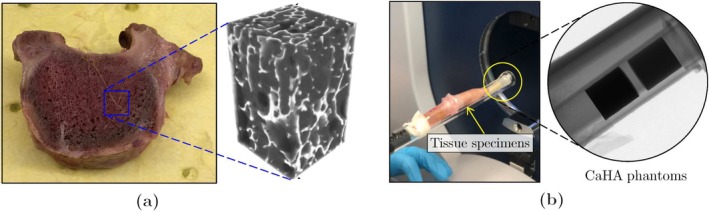
(a) Cadaveric vertebral sample and the micro‐QCT images associated with one of the cuboid specimens extracted from its trabecular region; (b) specimens setup and CaHA calibration phantoms within the micro‐QCT scanner tube.

Micro‐QCT scans of the trabecular bone tissue used in this study were prepared using a cadaveric specimen (Figure 1a). The autopsy subject was a 57‐year‐old female with no history of infectious disease. Five 6 mm × 6 mm × 10 mm cuboidal specimens, collected from various parts of the thoracic vertebra, were extracted in the principal direction from the cadaveric vertebra. The samples were scanned using a Bruker SkyScan micro‐QCT scanner (Model 1276, Bruker Corporation, Billerica, MA, USA), with a spatial resolution of 20 μm and an x‐ray voltage of 40 KV (Figure 1b). More details regarding the preparation and imaging of these samples are provided in our earlier work in [32].

The voxelated gray‐scale (GS) fields obtained from micro‐QCT imaging of the trabecular samples were converted to an equivalent BMD distribution, as shown in Figure 1a. During the scans, a pair of Bruker calibration phantoms with 0.25 and 0.75 $g/{\mathrm{cm}}^{3}$ concentrations of calcium hydroxyapatite (CaHA) were also scanned. The known mass concentrations of these phantoms were calibrated with their measured GS values from the micro‐QCT images, enabling the calibration of bone BMD based on the scanned GS values as

(1) ρ=α+βGS,

where α and β are calibration coefficients (evaluated as −5.53 and 0.013, respectively) and ρ is the bone density given in $\left[g/{\mathrm{cm}}^{3}\right]$. The DCGAN‐based ReconGAN framework introduced in reference [32] was implemented to virtually reconstruct larger, realistic microstructural models of the trabecular bone based on sample samples reconstructed from micro‐QCT data.

## 3 Theory

### 3.1 Internal Remodeling: BMD Changes

Several numerical models relying on FEM have been proposed to study bone remodeling, see for example [6, 7, 8, 9, 10, 16, 17, 23, 24]. In these models, a mechanically derived stimulus function based on measures such as the strain energy density (SED) or von Mises stresses drives the bone density to evolve towards a chosen threshold value, referred to as the attractor state stimulus [6]. In phenomenological models, the primary motivation is to satisfy Wolff's law, that is, to show the transformation of bone morphology or the re‐distribution of its mass follows the applied load. In other words, it is assumed that the bone adapts to an applied load such that the regions with higher values for the stimulus function end up having higher BMD values. Thus, phenomenological models only give a qualitative trend of density evolution. On the other hand, in mechanobiological models, researchers have generally used the difference between mechanical stimulus values and the corresponding threshold to drive the evolution of cell dynamics [16, 17], which is assumed to derive changes in bone density. Next, we briefly describe the model studied in this work, i.e., the mechanobiological model presented by Rapisarda et al. [17]. Following this, we also describe how we modify this model in this study.

#### 3.1.1 Mechanobiological Model Proposed by Rapisarda et al.

Rapisarda et al. proposed a mechanobiological approach to bone remodeling considering the interaction between osteoblasts, osteoclasts, and osteocytes [17, 33]. Osteocytes, which comprise 90%–95% of all bone cells, are former osteoblasts buried in the bone [34]. These cells are responsible for sensing mechanical stimuli and regulating bone formation and resorption [35]. Osteoclasts (1%–2% of bone cells) resorb old bone, while osteoblasts (4%–6% of bone cells) are responsible for replacing the old bone with new cells [36].

Based on these biological phenomena, the governing equations describing the evolution of cell densities and bone tissue density are given by

(2) $$\frac{\partial x_{k}}{\partial t}=-\beta_{k}X_{k}+\gamma_{\mathrm{bk}}x_{b}\kappa \left(\varphi \right),$$

(3) $$\frac{\partial x_{b}}{\partial t}=-\beta_{b}X_{b}-\gamma_{\mathrm{bk}}x_{b}\kappa \left(\varphi \right)+\alpha_{b}S^{+}x_{k},$$

(4) $$\frac{\partial x_{c}}{\partial t}=-\beta_{c}X_{c}+\gamma_{c}x_{c}\kappa \left(\varphi \right)+\alpha_{c}S^{-}x_{k},$$

(5) $$\frac{\partial \rho }{\partial t}=\left({\mathrm{ax}}_{b}-{\mathrm{bx}}_{c}\right)H\left(\varphi \right),$$

where ρ is the density of the bone tissue and $x_{k}$, $x_{b}$, and $x_{c}$ are the density of osteocytes, osteoblasts, and osteoclasts, respectively. Also, coefficients $\beta_{k}$, $\beta_{b}$, and $\beta_{c}$ denote apoptotic rates of the bone cells, $\gamma_{\mathrm{bk}}$ is the rate of conversion of osteoblasts to osteocytes, $\gamma_{c}$ is the differentiation rate of osteoclasts, and $\alpha_{b}$ and $\alpha_{c}$ are birth rates of osteoblasts and osteoclasts, respectively. The terms $X_{i}$ $\left(i=k,b,c\right)$ are threshold functions defined as

(6) $$X_{i}=\left\{\begin{array}{l} x_{i},\text{if}\;x_{i}>{\tilde{x}}_{i}\\ 0,\;\text{if}\;x_{i}\leq {\tilde{x}}_{i} \end{array}\right.,$$

where ${\tilde{x}}_{i}$ are threshold cell populations. $S^{+}$ and $S^{-}$ in (3) and (4) denote the positive and negative portions of the stimulus, responsible for the production of new osteoblasts and osteoclasts, respectively. The stimulus function can then be defined as

(7) $$S\left(x,t\right)=\left(\frac{\int_{B}U\left(y,t\right)\eta x_{k}\left(y,t\right)e^{-\frac{{\left\|x-y\right\|}^{2}}{D^{2}}}\mathrm{dy}}{\int_{B}e^{-\frac{{\left\|x-y\right\|}^{2}}{D^{2}}}\mathrm{dy}}\right)-S_{0}\left(x,t\right),$$

where B is the reference configuration, U is the strain energy density, η denotes how much the stimulus is affected by the density of osteocytes (assumed as η=1.0), D is the range of action of osteocytes, and $S_{0}$ is the reference stimulus value corresponding to a biological equilibrium state in which effects of resorption and formation are balanced.

#### 3.1.2 Evaluation of the Model by Rapisarda et al.

Next, we investigate the performance of the mechanobiological model proposed by Rapisarda et al. for simulating the bone remodeling process [17, 33]. In this study, we considered the hydrostatic equilibrium state wherein $S^{+}=S^{-}=0$, that is, the stimulus everywhere in the bone sample equals $S_{0}$. The goal is to determine whether this model can maintain mass equilibrium in this state, which corresponds to a state of biological equilibrium where the effects of resorption and formation are balanced. As this condition assumes spatial uniformity in the mechanical stimulus, no FE analysis was required. Instead, the governing system of ordinary differential equations (ODEs) describing cell population dynamics and density evolution was implemented and solved in MATLAB. In the remainder of this manuscript, we assume trabecular and cortical bones have a linear elastic behavior and $\kappa \left(\varphi \right)=H\left(\varphi \right)=1$ [17, 33]. The governing equations characterizing the variation of bone cells can then be simplified as

(8) $$\frac{\partial x_{k}}{\partial t}=-\beta_{k}X_{k}+\gamma_{\mathrm{bk}}x_{b},$$

(9) $$\frac{\partial x_{b}}{\partial t}=-\beta_{b}X_{b}-\gamma_{\mathrm{bk}}x_{b},$$

(10) $$\frac{\partial x_{c}}{\partial t}=-\beta_{c}X_{c}+\gamma_{c}x_{c},$$

(11) $$\frac{\partial \rho }{\partial t}=\left({\mathrm{ax}}_{b}-{\mathrm{bx}}_{c}\right).$$

Considering the case where $x_{b}\leq {\tilde{x}}_{b}$ and $x_{c}\leq {\tilde{x}}_{c}$, and thereby $X_{b}=X_{c}=0$ and also $\gamma_{c}=0$ [33], Then, governing equations determining the variation of OBs and OCs are reduced to

(12) $$\frac{\partial x_{b}}{\partial t}=-\gamma_{\mathrm{bk}}x_{b},$$

(13) $$\frac{\partial x_{c}}{\partial t}=0.$$

**FIGURE 2:**
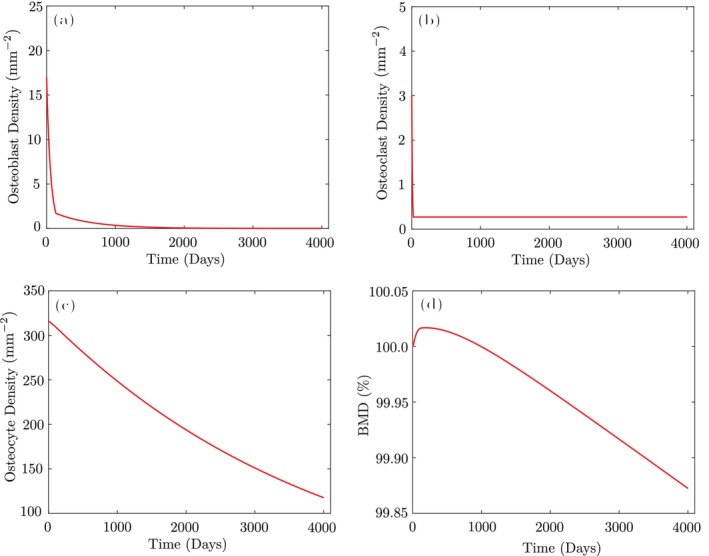
Rapisarda model non‐equilibrium in the hydrostatic state: (a) Osteoblasts; (b) osteoclasts; (c) osteocytes; (d) BMD.

The equation above means although $x_{b}$ keeps reducing below the threshold ${\tilde{x}}_{b}$, $x_{c}$, it does not reduce below ${\tilde{x}}_{c}$. Therefore, the bone keeps on resorbing, which implies a non‐equilibrium condition. This behavior of cells is illustrated in the plots in Figure 2a–c. As a result, the model proposed by Rapisarda et al. [17, 33] is unable to maintain bone mass in the hydrostatic equilibrium state (see Figure 2d), which has motivated modifying this model in this research work to remove this shortcoming.

#### 3.1.3 Mechanobiological Model Proposed in This Study

The model proposed in this study builds upon the mechanobiological model proposed by Rapisarda et al. [17, 33]. The major difference in our model is that we differentiate between active and inactive osteoblasts and osteoclasts and theorize that only active cells take part in the remodeling process. Also, in addition to osteoblasts, osteoclasts, and osteocytes, we consider the effects of inactive osteoclasts [37] and inactive osteoblasts (bone lining cells). The latter refers to post‐proliferative osteoblasts that are flat in shape and line the external surfaces of bone in a quiescent state [38].

Based on these biological phenomena, the system of ODEs describing the evolution of cell densities and bone tissue density is given by

(14) $$\frac{\partial x_{k}}{\partial t}=-\beta_{k}x_{k}+\gamma_{\mathrm{bk}}X_{b}\kappa \left(\varphi \right),$$

(15) $$\frac{\partial x_{b}}{\partial t}=-\beta_{b}X_{b}-\gamma_{\mathrm{bk}}X_{b}\kappa \left(\varphi \right)+\alpha_{b}S^{+}x_{k},$$

(16) $$\frac{\partial x_{c}}{\partial t}=-\beta_{c}X_{c}+\gamma_{c}X_{c}\kappa \left(\varphi \right)+\alpha_{c}S^{-}x_{k},$$

(17) $$\frac{\partial \rho }{\partial t}=\left({\mathrm{aX}}_{b}-{\mathrm{bX}}_{c}\right)H\left(\varphi \right),$$

where $x_{i}$ $\left(i=k,b,c\right)$, ρ, $\beta_{k}$ $\left(i=k,b,c\right)$, $\gamma_{\mathrm{bk}}$, $\gamma_{c}$, $\alpha_{b}$, and $\alpha_{c}$ retain the same meaning as in the mechano‐biological model proposed by Rapisarda et al. as detailed in Section 3.1.1. However, the primary distinction between the models lies in the interpretation of $X_{i}$ $\left(i=b,c\right)$. While we do not differentiate between active and inactive osteocytes–due to a lack of evidence in the literature–we do distinguish between active and inactive osteoblasts and osteoclasts. This differs from Rapisarda et al., who define threshold functions for all bone cells in Equation (6), which are not the same as defining active and inactive cells. In our model, $X_{i}$ $\left(i=b,c\right)$ refers specifically to the active bone cells, and is defined as follows:

(18) $$X_{i}=\left\{\begin{array}{c} x_{i}-{\tilde{x}}_{i},\text{if}\;x_{i}>{\tilde{x}}_{i}\\ 0,\;\text{if}\;x_{i}\leq {\tilde{x}}_{i} \end{array}\right..$$

Here, ${\tilde{x}}_{i}$ represents inactive osteoclasts for $\left(i=c\right)$ and bone lining cells for $\left(i=b\right)$, respectively. These inactive cells do not participate directly in bone remodeling, so they are subtracted from the total number of cells to isolate the active cells that contribute to the remodeling process. Furthermore, $S^{+}$ and $S^{-}$ denote the positive and negative parts of the stimulus, similar to the mechanobiological model proposed by Rapisarda et al. [17, 33], though the formulation of the stimulus in our model has been modified and can be written as

(19) $$S_{\text{total}}\left(x,t\right)={\left(\sum_{i=1}^{N}n_{i}{\left(S_{i}\left(x,t\right)\right)}^{m}\right)}^{1/m}-S_{0}\left(x,t\right),$$

where $S_{i}\left(x,t\right)$ is considered to be the stimulus for one load cycle, constituting the total stimulus $S_{\text{total}}\left(x,t\right)$ over N load cycles. Also, $n_{i}$ is the number of load cycles per day for load i, and m is a model parameter determining how the stimulus affects the rate of bone mass loss/gain. Note that, in Equation (19), $S_{i}\left(x,t\right)$ is evaluated similar to Equation (7) following Rapisarda et al. [17, 33], that is,

(20) $$S_{i}\left(x,t\right)=\left(\frac{\int_{B}U\left(y,t\right)\eta x_{k}\left(y,t\right)e^{-\frac{{\left\|x-y\right\|}^{2}}{D^{2}}}\mathrm{dy}}{\int_{B}e^{-\frac{{\left\|x-y\right\|}^{2}}{D^{2}}}\mathrm{dy}}\right).$$

With this formulation, we directly incorporate the spatial distribution of osteocytes, ensuring that mechanical stimuli are only effective in regions where sufficient osteocytes are present to detect them. If the osteocyte density $x_{k}\left(y,t\right)$ is sparse or absent in a given neighborhood, the corresponding stimulus $S_{i}\left(x,t\right)$ is diminished, irrespective of the magnitude of the mechanical load $U\left(y,t\right)$. Thus, the model explicitly couples the mechanical environment to local cellular activity, making the stimulus field responsive to both spatial and temporal variations in osteocyte distribution. This coupling is particularly crucial in patient‐specific applications, where subtle differences in cell populations and stress distributions can substantially influence the evolution of BMD.

To simulate the remodeling process numerically, the system of equations was implemented using the Abaqus Python scripting interface. The governing equations were solved iteratively, and the spatially varying BMD predictions were updated through custom scripts that interfaced with Abaqus FE simulations, enabling efficient integration of the mechanobiological model into the computational workflow.

#### 3.1.4 Parameter Calibration Under Hydrostatic Conditions

Before analyzing the performance of the modified mechanobiological model proposed in this work, it is crucial to calibrate parameters such as $\beta_{k}$, $\beta_{b}$, $\beta_{c}$, $\gamma_{\mathrm{bk}}$, $\gamma_{c}$, $\alpha_{b}$, $\alpha_{c}$, a and b used in the cells governing Equations ((2), (3), (4), (5)) under hydrostatic (this section) and non‐hydrostatic (next section) conditions. Note that mature osteoclasts are terminally differentiated cells and do not undergo mitosis, that is, we can set $\gamma_{c}=0$ [33]. The remaining parameters must be calibrated such that the model can maintain the mass equilibrium under hydrostatic conditions $\left(S^{+}=S^{-}=0\right)$, where Equations ((2), (3), (4), (5)) reduce to

(21) $$\frac{\partial x_{k}}{\partial t}=-\beta_{k}x_{k}+\gamma_{\mathrm{bk}}X_{b}\kappa \left(\varphi \right),$$

(22) $$\frac{\partial x_{b}}{\partial t}=-\beta_{b}X_{b}-\gamma_{\mathrm{bk}}X_{b}\kappa \left(\varphi \right),$$

(23) $$\frac{\partial x_{c}}{\partial t}=-\beta_{c}X_{c}+\gamma_{c}X_{c}\kappa \left(\varphi \right),$$

(24) $$\frac{\partial \rho }{\partial t}=\left({\mathrm{aX}}_{b}-{\mathrm{bX}}_{c}\right)H\left(\varphi \right).$$

According to the equations above, in the hydrostatic state, we only need to calibrate some of the model parameters, that is, $\beta_{k}$, $\beta_{b}$, $\beta_{c}$, $\gamma_{\mathrm{bk}}$, $\gamma_{c}$, a, and b. Again, since the hydrostatic condition assumes spatial uniformity in the mechanical stimulus (i.e., $S^{+}=S^{-}=0$), no FE analysis was required. Instead, the ODEs characterizing cell population dynamics and density evolution was implemented and solved in MATLAB, similar to Section 3.1.2. Here, we propose an optimization‐based strategy relying on the Genetic Algorithm (GA) [39] for tuning these parameters with the objective function of minimizing the BMD change Δρ resulting in the conservation of mass under homeostasis/equilibrium. GA is an evolutionary optimization algorithm starting by generating a random initial population, which in this work comprises various initial values of $\beta_{k}$, $\beta_{b}$, $\beta_{c}$, $\gamma_{\mathrm{bk}}$, $\gamma_{c}$, a, and b, all selected within a permissible range. After encoding each parameter into a string of binaries (chromosomes), the optimization process begins by evolving this initial population into a more optimized configuration (chromosomes with higher fitness) using genetic operator selection, cross‐over, and mutation.

To perform GA optimization, we need to provide meaningful ranges for different parameters used in the model based on literature data. It is known that osteoblasts at the end of the remodeling cycle have one of the following fates: (1) become embedded in the bone matrix and differentiate into osteocytes, (2) become quiescent bone lining cells, or (3) die by apoptosis [40]. It has been reported that, in human cancellous bone, around 65% of osteoblasts undergo apoptosis and about 30% transform into osteocytes [41], meaning only about 5% of them become quiescent bone lining cells. We can estimate the value of $\gamma_{\mathrm{bk}}$ (rate of osteoblasts‐to‐osteocytes differentiation) based on this observation and the fact that osteoblasts have an average lifespan of 3 months. Therefore, under hydrostatic conditions (in the absence of osteoblast production), it is assumed that 30% osteoblasts transform into osteocytes within 3 months, that is,

(25) $${\left(1-\gamma_{\text{bk}}\right)}^{90}=0.70,$$

which yields $\gamma_{\mathrm{bk}}=0.004$.

Similarly, it is known that osteocytes have an average lifespan of 25 years and some can live up to 50 years [42]. Because bone remodeling occurs within a few months (120 days for cortical bone and 200 days for trabecular bone), we cannot estimate the death rate of osteocytes via GA optimization, as their lifespan (25–50 years) is much higher than that of osteoclasts (3 weeks) and osteoblasts (3 months). Instead, assuming that the half‐life of osteocytes is about 10 years (3650 days), the value of $\beta_{k}$ can be estimated as

(26) $$x_{k}\left(3650\right)=\frac{x_{k0}}{2}=x_{k0}e^{-3650\beta_{k}},$$

which yields $\beta_{k}=1.9e-04$.

The existence of two types of osteoclast cells has been reported in the literature: active and inactive [37]. Osteoclasts with a ruffled border are considered active, and those without the ruffled border are considered inactive [37]. In our model, similar to inactive osteoblasts (bone lining cells) and based on the data reported in [37], it is assumed that 5% of osteoclasts are inactive.

Next, we must define meaningful ranges for the values of the bone formation rate a and the bone resorption rate b. Gruber et al. [43] studied the quantitative histology of trabecular bone in human iliac crest bone biopsies and reported the osteoblast activity to be 2.9e−05±8.8e−06 ${\mathrm{mm}}^{2}/\mathrm{day}$ and the osteoclast activity to be 2.6e−04±1.0e−04 ${\mathrm{mm}}^{2}/\mathrm{day}$. In (24), since the left‐hand‐side is given in $g/{\mathrm{mm}}^{3}/\mathrm{day}$, the unit of the bone formation/resorption rates a and b should be ${\mathrm{mm}}^{3}/\mathrm{day}$ ($X_{i}$ is given in mm^−3^). Hence, we multiply the osteoblast/osteoclast activity by the trabecular lamellar thickness taken as 6 μm [44, 45].

We also need meaningful ranges for the apoptotic rates of osteoblasts and osteoclasts ($\beta_{b}$ and $\beta_{c}$) to initiate the GA optimization. The average lifespans of osteoclasts and osteoblasts are 3 weeks and 3 months, respectively, which are comparable to the remodeling cycle period (120 days for cortical bone and 200 days for trabecular bone). Therefore, unlike the value of $\beta_{k}$, the model is very sensitive to the values of these parameters. Note that, the governing equations given in ((2), (3), (4), (5)) yield exponential solutions for the variation of $x_{b}$ and $x_{c}$, meaning the cell population reduces much faster early on and then its rate of change decreases. Therefore, assuming the half‐lives of these cells to be half of their average lifespan (3 weeks and 3 months) is rather unrealistic. Instead, the half‐lives of osteoclasts and osteoblasts are assumed to be in the range of 10% to 30% of their lifespans. Therefore, we assume that the values of $\beta_{b}$ and $\beta_{c}$ are in the range of $\left[0.023,0.069\right]$ and $\left[0.116,0.347\right]$, respectively.

The GA optimization under hydrostatic conditions aimed at preserving the trabecular bone mass using the initial values of parameters within the ranges discussed above yielded the optimized values of osteoblast and osteoclast activities to be 2.3044e−5 ${\mathrm{mm}}^{2}/\mathrm{day}$ and 2.5454e−4 ${\mathrm{mm}}^{2}/\mathrm{day}$, respectively. To calculate the optimized values of bone formation/resorption rates, these values are multiplied by the trabecular lamellar thickness (6 μm [46]), which yields a=1.3826e−7 ${\mathrm{mm}}^{3}/\mathrm{day}$ and b=1.5272e−6 ${\mathrm{mm}}^{3}/\mathrm{day}$. The optimal values of the remaining parameters using this optimization‐based approach are estimated as $\beta_{b}=0.030685$ and $\beta_{c}=0.13203$. A similar process is repeated for cortical bone, resulting in a=1.4294e−07 ${\mathrm{mm}}^{3}/\mathrm{day}$, b=2.0404e−06 ${\mathrm{mm}}^{3}/\mathrm{day}$, $\beta_{b}=0.040646$, and $\beta_{c}=0.20146$.

**FIGURE 3:**
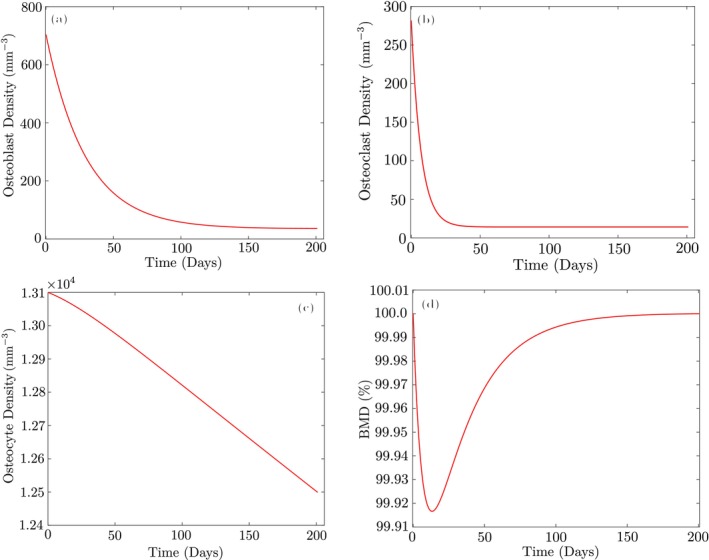
Behavior of the proposed model for trabecular bone in the hydrostatic state: (a) Osteoblasts; (b) osteoclasts; (c) osteocytes; (d) BMD.

Since osteoblast/osteoclast formation terms do not participate in ((21), (22), (23), (24)) in the hydrostatic state, all active osteoblast and osteoclast cells must die at the end of the trabecular bone remodeling cycle (about 200 days) [47] and only inactive cells remain. As shown in Figure 3, after the GA optimization, the proposed model maintains bone mass under hydrostatic conditions in the trabecular bone $\left(S^{+}=S^{-}=0\right)$. The plots for cortical bone are nearly identical and therefore not repeated here. Moreover, most active cells die around their average lifespan, which is 3 months for osteoblasts and 3 weeks for osteoclasts. This validates the correctness of the proposed model and demonstrates its ability to reproduce the expected behavior under hydrostatic conditions. Also, the GA optimization under hydrostatic conditions was executed on a system equipped with a 2.3 GHz Intel Core i5 processor (4 physical cores, 8 logical threads). MATLAB's parallel computing toolbox was utilized, and the optimization converged in approximately 10 s.

#### 3.1.5 Parameter Calibration Under Non‐Hydrostatic Conditions

Given the simplified version of the cells governing equation in the hydrostatic condition, the model parameters estimated in Section 3.1.4 do not include $\alpha_{b}$ and $\alpha_{c}$. Calibrating these parameters requires applying a different loading on the bone to simulate a non‐hydrostatic condition $\left(S^{+}\neq 0,S^{-}\neq 0\right)$. Using an optimization‐based approach, the primary objective function is once again the minimization of Δρ (conservation of mass). However, in non‐hydrostatic conditions, active bone cells do not die at the end of the remodeling cycle due to the presence of non‐zero cell production terms $\alpha_{b}S^{+}x_{k}$ and $\alpha_{c}S^{-}x_{k}$ in ((2), (3), (4), (5)). It is reported that, under bone homeostasis in healthy human bone, the ratio of different bone cells is maintained in the range of 90%–94% for osteocytes, 4%–6% for osteoblasts, and 0%–2% for osteoclasts [33, 36]. Therefore, in addition to the minimization of Δρ, we provide the ranges of bone cell ratios at homeostasis as constraints to the optimizer.

Since only two parameters ($\alpha_{b}$ and $\alpha_{c}$) must be estimated under non‐hydrostatic conditions, here we implement the *interior‐point algorithm* to tackle this constrained minimization problem. For a given optimization problem $\min_{x}f\left(x\right)$ subject to constraints $h\left(x\right)=0$ and $g\left(x\right)\leq 0$, all equality constraints are used to transform the problem using positive slack variables $s_{i}$ such that

(27) $$g\left(x\right)+s=0.$$

Using μ>0, the optimization problem is then approximated as

(28) $$\min_{x,s}f_{\mu }\left(x,s\right)=\min_{x}f\left(x\right)-\mu \sum_{i}\ln\left(s_{i}\right),$$

subject to s≥0, $h\left(x\right)=0$, and $g\left(x\right)+s=0$. The number of slack variables $s_{i}$ corresponds to the number of inequality constraints g. As μ approaches zero, the minimum of $f_{\mu }$ in the equation above approaches the minimum of f.

Using the optimized values of $\beta_{k}$, $\beta_{b}$, $\beta_{c}$, $\gamma_{\mathrm{bk}}$, $\gamma_{c}$, a and b previously calculated in Section 3.1.4, (28) is employed to estimate the values of $\alpha_{b}$ and $\alpha_{c}$ under non‐hydrostatic conditions. Here, we consider a realistic loading scenario based on the ground reaction forces (GRF) data corresponding to walking reported in [48], using a standard deviation of 7.5% for the average coefficient of variation (CV). The average compressive force on the L3–L4 vertebral segment during walking is reported as 1.0 times the body weight (BW) [49]. In a vertebral body, it is estimated that the cortical shell and the trabecular region carry 45% and 55% of an applied compressive load, respectively [50]. For a human subject weighting 75kg and an average cross‐sectional area of $1126\;{\mathrm{mm}}^{2}$ [51] for the lumbar spine, the average walking load on the trabecular and cortical bones are estimated to be 0.3594MPa and 0.2940MPa, respectively.

To estimate the value of $S_{0}\left(x,t\right)$, note that the threshold or attractor state stimulus (minimum amount of stimulus necessary to maintain bone mass) is given by

(29) $$S_{0}\left(x,t\right)=n_{0}^{\left(1⁄m\right)}U_{0}x_{\text{k}}\left(y,t\right),$$

where $n_{0}$ is the number of cycles of the applied load in the equilibrium state and $U_{0}$ is the SED threshold evaluated as

(30) $$U_{0}=P^{2}\left(1+\nu_{0}\right)\left(1-2\nu_{0}\right)/2E_{0}\left(1-\nu_{0}\right).$$

In the equation above, P is the applied compressive load in MPa, $E_{0}$ is the elastic modulus, and $\nu_{0}$ is the Poisson's ratio.

According to [52], we assume that trabecular and cortical bones with apparent densities ${\left(\rho_{0}\right)}_{\text{trab}}=0.225\;g/{\mathrm{cm}}^{3}$ and ${\left(\rho_{0}\right)}_{\text{cort}}=1.20\;g/{\mathrm{cm}}^{3}$, respectively, maintain their mass after $n_{0}=5000$ cycles of walking load. The elastic modulus of the trabecular bone (in GPa) can be evaluated as [53]

(31) $$E_{\text{trab}}=-0.16+0.004\rho +1.1e-06\rho^{2},$$

where ρ is in $g/{\mathrm{mm}}^{3}$. For cortical bone, we use the following empirical relation to calculate the elastic modulus as [54]

(32) $$E_{\text{cort}}=6.40\rho^{1.54},$$

where $E_{\text{cort}}$ is in GPa and ρ is in $g/{\mathrm{cm}}^{3}$. The Poisson's ratios for trabecular and cortical bones are estimated as 0.2 and 0.3, respectively. Also, the parameter m in (29) and (19) is taken as 4 based on the reported range of 2.3−4.8 by Whalen et al. [55]. Note that Beaupre et al. [6] also used m=4 in their work.

**FIGURE 4:**
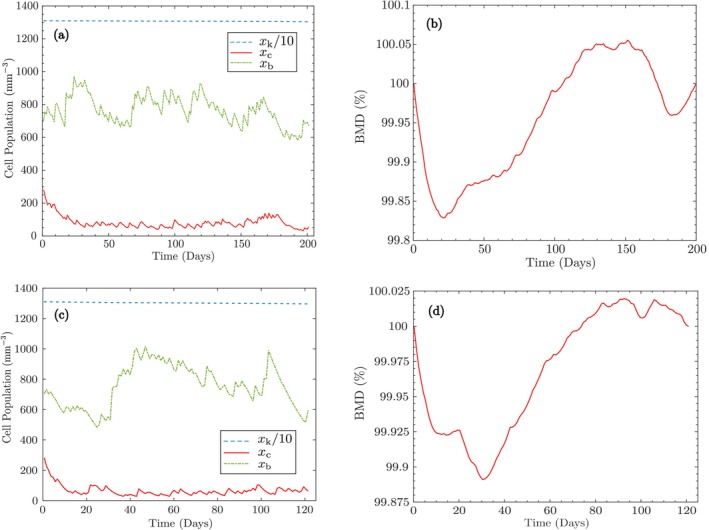
Proposed model maintains bone mass in the non‐hydrostatic state for trabecular and cortical bones: (a, c) Cell populations of osteocytes $x_{k}$, osteoblasts $x_{b}$, and osteoclasts $x_{c}$; (b, d) BMD variation as a percentage of initial BMD.

After performing the optimization using the parameter values/ranges reported above, the optimized values of $\alpha_{b}$ and $\alpha_{c}$ for the trabecular bone are obtained as 3.92e−3 and 1.38e−3, respectively. The corresponding values for the cortical region are evaluated as 9.313e−2 and 3.068e−2, respectively. Figure 4 illustrates the variation of bone cells and BMD for the optimized values, showing the capability of the proposed model to maintain bone mass in the non‐hydrostatic state. Also, the optimization using the interior‐point algorithm under non‐hydrostatic conditions was executed in serial on a 2.3 GHz Intel Core i5 processor (4 cores, 8 threads) and completed in approximately 1 min.

### 3.2 Surface Remodeling and Volume Change

Most studies on bone remodeling focus on tracking the changes in BMD without explicitly capturing the evolution of bone surface morphology. Here, we extend our mechanobiological model (Section 3.1.3) to estimate the change in apparent BMD $\Delta \rho_{\mathrm{app}}$ at the macroscale level. This change is then related to porosity to track volume fraction variations at the microstructural scale.

At the macroscale, the apparent density $\rho_{\mathrm{app}}$ is linked to porosity n as

(33) $$n=1-\frac{\rho_{\mathrm{app}}}{\rho_{t}},$$

where $\rho_{t}$ is the tissue density. In our model, it is assumed that during bone remodeling, only the apparent density $\rho_{\mathrm{app}}$ and porosity n are changing, meaning the tissue density $\rho_{t}$ remains constant. We thus differentiate between a macroscale “equivalent continuum” description $\rho_{\mathrm{app}}$ and its corresponding porosity n, reflecting microstructural void space. Subsequently, the ratio of the initial to final bone volume fractions $\left(\mathrm{BV}/\mathrm{TV}\right)$ may be written as

(34) $$f=\frac{{\left(\frac{\mathrm{BV}}{\mathrm{TV}}\right)}_{f}}{{\left(\frac{\mathrm{BV}}{\mathrm{TV}}\right)}_{i}},$$

where porosity n and bone volume fraction $\left(\mathrm{BV}/\mathrm{TV}\right)$ are related as

(35) $$n=1-\frac{\mathrm{BV}}{\mathrm{TV}}.$$

At the microscale, we employ the ABAQUS TOSCA Shape Optimization module [56] to simulate surface (morphological) changes by imposing the volume‐fraction ratio f as a constraint. The optimization then seeks to minimize the maximum von Mises stress $\sigma_{\mathrm{vm}}$ over the microscale domain. While other measures like strain energy density (SED) or non‐uniformity of stress/strain are common in the literature for remodeling‐based optimization, Tsubota and Adachi [57] demonstrated that minimizing the non‐uniformity of von Mises stress can lead to more uniform stress distributions, thereby motivating our choice of $\sigma_{\mathrm{vm}}$.

**FIGURE 5:**
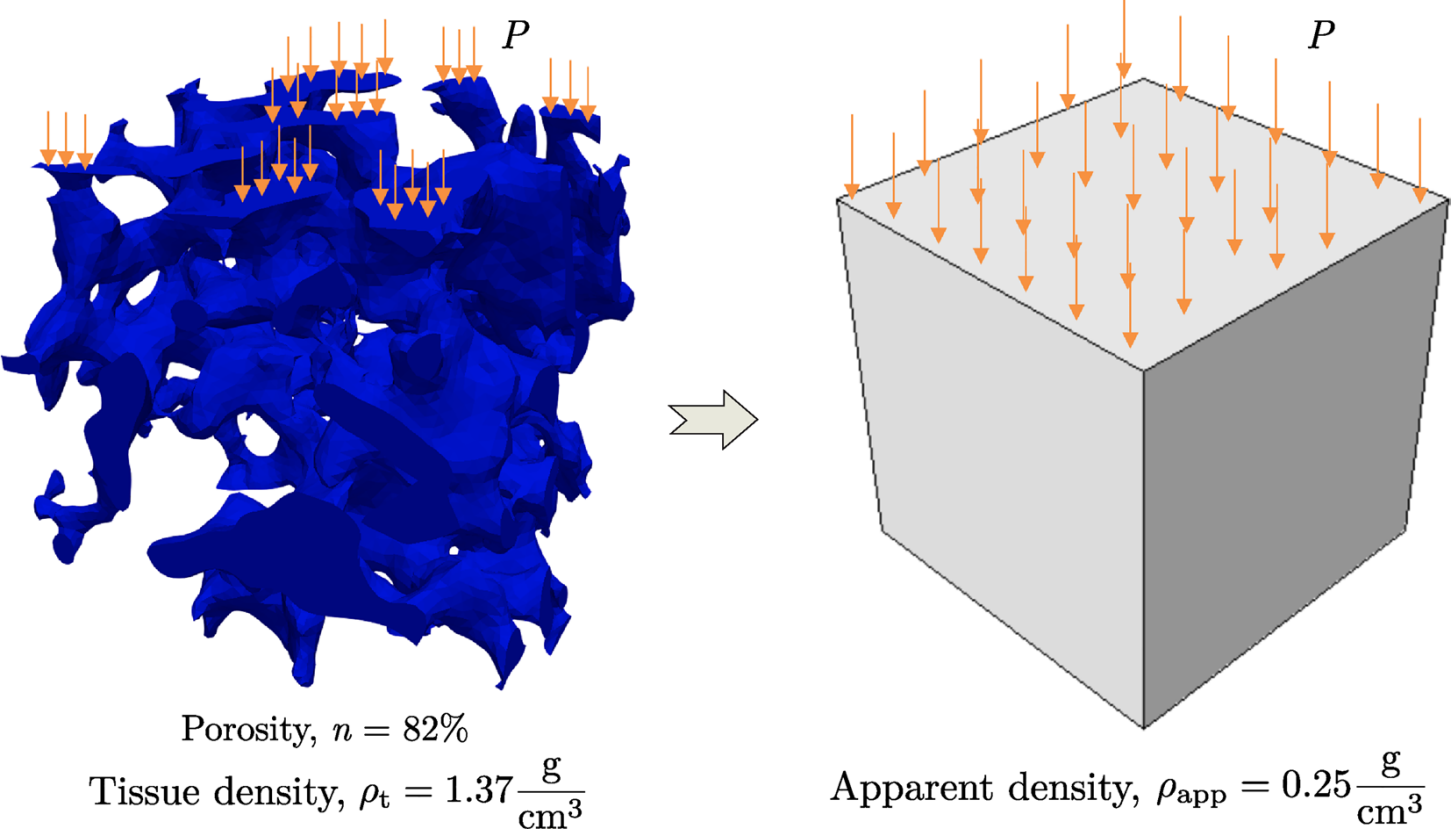
Equivalent macroscale model with apparent density ${\left(\rho_{\mathrm{app}}\right)}_{\text{trab}}$ for a given trabecular bone microstructural model with porosity n and tissue density ${\left(\rho_{t}\right)}_{\text{trab}}$ subject to compressive load P.

To summarize the workflow, Figure 5 illustrates how the macroscale model (using $\rho_{\mathrm{app}}$ to represent an “equivalent continuum”) is linked to the trabecular microstructure with porosity n and tissue density $\rho_{t}$. The mechanobiological model in Section 3.1.3 governs changes in $\rho_{\mathrm{app}}$ at the macroscale, whereas the shape optimization at the microscale refines local morphological features to minimize von Mises stress for the specified volume‐fraction change. This dual‐scale approach ensures that the global (macroscale) bone adaptation remains consistent with the local (microscale) morphological evolution, thus offering a more holistic representation of patient‐specific bone remodeling behavior.

All compressive loads, their literature origins, and the stochastic variability used in the model are summarized in Table 1. This table demonstrates the differences between the loading associated with the ISS micro‐gravity phase, prolonged bed rest, and the various rehabilitation activities modelled later in Section 4.

**TABLE 1:** Vertebral $\left(L_{3}–L_{4}\right)$ compressive loads used in the simulations.

Scenario	Activity/phase	Trabecular (MPa)	Cortical (MPa)	Source	Variability (SD/CV)
Earth baseline/adaptation	Walking (8000 steps/day)	0.36	0.29	[49]	CV 7.5% [48]
ISS (micro‐gravity)	Walking (1280 steps/day)	0.27	0.22	[58]	CV 7.5% [48]
Bed rest	Supine lying	0.054	0.044	[59]	None (constant)
Rehabilitation	Walking (10,000 steps/day)	0.36	0.29	[49]	CV 7.5% (assumed)
	Running (8 mph)	0.54	0.44	[48]	CV 7.5% (assumed)
	Jumping	1.07	0.87	[48]	CV 7.5% (assumed)
	Body‐weight squat	0.63	0.52	[60]	CV 7.5% (assumed)

*Note:* Loads are partitioned 55% to trabecular bone and 45% to cortical bone.

Abbreviations: BW = body‐weight multiple; CV = coefficient of variation (assumed unless specified).

## 4 Results and Discussion

### 4.1 Preliminary Validation of the Proposed Model

In this section, we implement several real‐life datasets available in the literature for preliminary validation of the modified mechanobiological model proposed in this work. The data used in this study include both controlled and uncontrolled experimental data to demonstrate that this model can predict a realistic rate of bone apposition and resorption in different case scenarios. The uncontrolled datasets used in this study include the bone loss and recovery in astronauts [58, 61, 62, 63] and during prolonged bed rest [64]. Since not all data required for creating the model (e.g., exact activities taken by the subject) are reported, we needed to make several assumptions during the modeling process. Therefore, we also used two controlled datasets reported by Iwamoto et al. [65] and Yamazaki et al. [52] for the percentage of change in apparent BMD for different activity levels for this preliminary validation study. A detailed sensitivity analysis was performed on the performance of our model based on the data reported in reference [52] to determine if predicted BMD values are within the range of experimentally measured data. In this sensitivity analysis, we used different combinations of model parameters ($\beta_{k}$, $\beta_{b}$, $\beta_{c}$, $\gamma_{\mathrm{bk}}$, $\gamma_{c}$, $\alpha_{b}$, $\alpha_{c}$, a, and b), which could vary in different individuals and is essential to build patient‐specific models in the future.

#### 4.1.1 BMD Loss and Recovery in Astronauts

Astronauts going on long‐duration space missions in the International Space Station (ISS) significantly lose bone mass on return to Earth; hence, they often undergo special rehabilitation programs to recover bone strength afterward [62, 63]. This can be attributed to the reduced activity levels (for instance, walking steps/day and time spent standing) and reduced gravity that lowers average loads applied to bone [58]. Here, we use available data on bone loss and recovery in astronauts as a real‐world example for the preliminary validation of the mechanobiological model introduced in this work for predicting the remodeling process.

The main physical activity undertaken by astronauts on ISS is walking, which can be used to estimate the net loaded time on bone. Cavanagh et al. [58] report that the net loaded time of all activities in a day (stand, walk, run, and other) decreased by 84% on ISS compared to similar activities on earth. Assuming 8000 walking steps/day to be typical for an astronaut on earth, 16% of that (1280 steps/day) could be considered as the typical activity on ISS. Moreover, Cavanagh et al. [58] studied the average foot forces during walking on earth and ISS, which was reported to be 1.18BW and 0.89BW, respectively (BW: body weight). For an astronaut weighing 75kg, load due to BW is 0.65MPa. Also, Cappozzo and Aurelio [49] reported that the average compressive force on the L3–L4 vertebral segment during walking is BW. Once again, assuming that this force is shared between the trabecular and cortical regions at 55% to 45% ratio, the corresponding loads in each region would be 0.36MPa and 0.29MPa on earth, respectively. Using the ratio of 1.18 to 0.89 between earth and ISS forces, as reported in reference [58], the corresponding walking loads on ISS would be 0.27MPa and 0.22MPa, respectively. In this example, we also assume some variability with a maximum standard deviation of 7.5% [48] in the walking load.

The validation study was carried out in three stages:

- Stage I: Macroscopic bone adaptation to walking loads on Earth
- Stage II: BMD loss in space
- Stage III: BMD recovery post‐return to Earth

A $4\;{\mathrm{mm}}^{3}$ trabecular bone FE model was generated using Simpleware ScanIP. The tissue density $\left(\rho_{t}\right)$ was estimated using a volume‐averaged approach as

(36) $$\rho_{t}=\frac{\sum_{i=1}^{N_{e}}\rho_{i}V_{i}}{\sum_{i=1}^{N_{e}}V_{i}},$$

where $N_{e}$ is the number of elements in the FE mesh, and $\rho_{i}$ and $V_{i}$ are the element‐wise density and volume. This gives ${\left(\rho_{t}\right)}_{\text{trab}}=1.368\;g/{\mathrm{cm}}^{3}$ and trabecular bone porosity estimated using Simpleware ScanIP is 82%. Using (33), the apparent density is ${\left(\rho_{\mathrm{app}}\right)}_{\text{trab}}=0.246\;g/{\mathrm{cm}}^{3}$. The elastic modulus was calculated using (31), and the macroscale model was constructed as shown in Figure 5.

For the VCF simulations in Section 4.3 [66], the vertebra was modeled with a cortical shell having an elastic modulus of 10GPa. According to (32), the corresponding apparent density is estimated as ${\left(\rho_{\mathrm{app}}\right)}_{\text{cort}}=1.337g/{\mathrm{cm}}^{3}$. Based on Osterhoff et.al. [67], we assume a porosity of 2.5% to calculate the tissue density as ${\left(\rho_{t}\right)}_{\text{cort}}=1.371g/{\mathrm{cm}}^{3}$ using (33).

Next, the macroscopic bone adaptation for trabecular and cortical bones we simulated assuming 8000 walking steps/day, using walking loads of 0.36MPa and 0.29MPa, respectively. The convergence criterion is set when the BMD changes over the last 100 days is less than $1\times 10^{-3}$, which can be written as

(37) $$100\times \frac{\left(\rho_{i}-\rho_{i-100}\right)}{\rho_{i}}<0.001.$$

**FIGURE 6:**
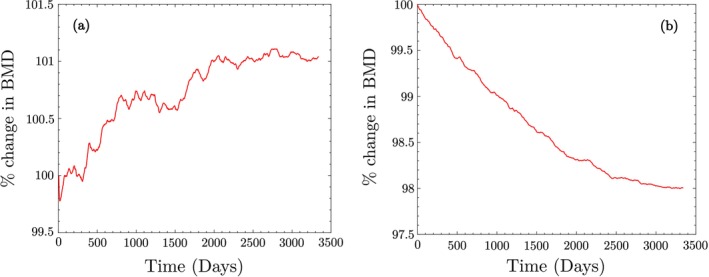
Change in apparent BMD as a percentage of initial BMD during the adaptation of macroscale bone to 8000 steps/day of walking in trabecular and cortical bones.

The variation in BMD during macroscopic bone adaptation for both trabecular and cortical bones is shown in Figure 6. After adaptation, the apparent BMD values are ${\left(\rho_{\mathrm{app}}\right)}_{\text{trab}}=0.249g/{\mathrm{cm}}^{3}$ and ${\left(\rho_{\mathrm{app}}\right)}_{\text{cort}}=1.31g/{\mathrm{cm}}^{3}$. The final cell populations for trabecular bone are ${\left(x_{k}\right)}_{\text{trab}}=11300{\mathrm{mm}}^{-3}$, ${\left(x_{b}\right)}_{\text{trab}}=423{\mathrm{mm}}^{-3}$, and ${\left(x_{c}\right)}_{\text{trab}}=71{\mathrm{mm}}^{-3}$, while for cortical bone, they are ${\left(x_{k}\right)}_{\text{cort}}=7589{\mathrm{mm}}^{-3}$, ${\left(x_{b}\right)}_{\text{cort}}=272{\mathrm{mm}}^{-3}$, and ${\left(x_{c}\right)}_{\text{cort}}=19{\mathrm{mm}}^{-3}$. These values are used as initial conditions for Stage II.

**FIGURE 7:**
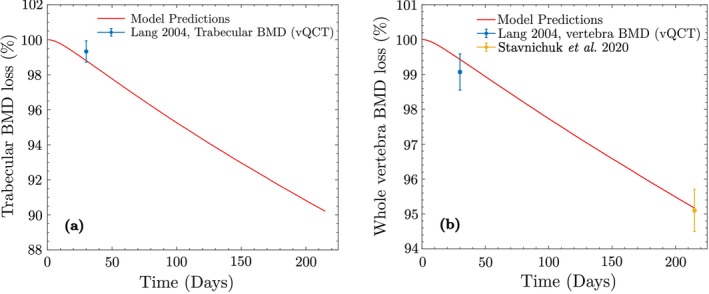
BMD loss in space travel, showing the change in apparent BMD as a percentage of the adapted BMD predicted using the proposed model, due to reduced activity level in space travel: (a) Trabecular bone; (b) whole vertebra.

Assuming that the astronaut begins the mission with the adapted bone properties, we proceed to simulate Stage II to approximate BMD loss during space travel. The mission duration was estimated as 215 days, based on the average data from Stavnichuk et al. [61]. Due to reduced activity in space, we assume 1280 walking steps/day with ISS walking loads of 0.27MPa for trabecular bone and 0.22MPa for cortical bone. Figure 7 shows the simulated BMD loss for both bone types and the whole vertebra. For the trabecular BMD value reported by Lang (Figure 7a), the model underpredicts the experimental measurement by approximately 0.6% (SD = ±0.7%). For the whole vertebra BMD (Figure 7b), the model overpredicts the Lang measurement by 0.3% (SD = ±0.5%) and overpredicts the Stavnichuk et al. value by 0.1% (SD = ±0.6%). In all cases, the model predictions lie within the reported standard deviations, indicating an excellent agreement with experimental measurements and falling well within clinically acceptable bounds. The final cell populations after space travel are ${\left(x_{k}\right)}_{\text{trab}}=10881{\mathrm{mm}}^{-3}$, ${\left(x_{b}\right)}_{\text{trab}}=36{\mathrm{mm}}^{-3}$, and ${\left(x_{c}\right)}_{\text{trab}}=469{\mathrm{mm}}^{-3}$ for trabecular bone, and ${\left(x_{k}\right)}_{\text{cort}}=7292{\mathrm{mm}}^{-3}$, ${\left(x_{b}\right)}_{\text{cort}}=35{\mathrm{mm}}^{-3}$, and ${\left(x_{c}\right)}_{\text{cort}}=167{\mathrm{mm}}^{-3}$ for cortical bone.

The BMD loss for the vertebra is estimated as a weighted average of the trabecular and cortical BMDs, weighted by their respective volume fractions. We assume a trabecular mass fraction of 75%, based on findings that trabecular bone accounts for 81% of total vertebra mass in men and 71% in women [68], that is,

(38) $$\frac{{\left(\rho_{t}\right)}_{\text{trab}}V_{\text{trab}}}{{\left(\rho_{t}\right)}_{\text{trab}}V_{\text{trab}}+{\left(\rho_{t}\right)}_{\text{cort}}V_{\text{cort}}}=0.75,$$

where ${\left(\rho_{t}\right)}_{\text{trab}}$ and ${\left(\rho_{t}\right)}_{\text{cort}}$ are the tissue densities of trabecular and cortical bones, and $V_{\text{trab}}$ and $V_{\text{cort}}$ are their respective volumes. The objective is to estimate the volume fractions of trabecular $\left(v_{\text{trab}}\right)$ and cortical $\left(v_{\text{cort}}\right)$, subject to the constraint $v_{\text{trab}}+v_{\text{cort}}=1$. These volume fractions are given by

(39) $$v_{\text{trab}}=\frac{V_{\text{trab}}}{V_{\text{trab}}+V_{\text{cort}}},\;\text{and}\;v_{\text{cort}}=\frac{V_{\text{cort}}}{V_{\text{trab}}+V_{\text{cort}}}.$$

In this study, it is assumed that the tissue density remains constant during the remodeling process, affecting only the apparent density and porosity. Therefore, using ${\left(\rho_{t}\right)}_{\text{trab}}=1.368g/{\mathrm{cm}}^{3}$ and ${\left(\rho_{t}\right)}_{\text{cort}}=1.371g/{\mathrm{cm}}^{3}$ and substituting them in (38) yields $v_{\text{trab}}=75.05\%$ and $v_{\text{cort}}=24.95\%$.

Finally, we simulate BMD recovery after returning to Earth (Stage III). The cell populations are initialized using the values at the end of Stage II to evaluate bone mass recovery under various loading scenarios. We begin with two walking regimens: 8000 steps/day and 10,000 steps/day. The third scenario includes a 45‐day rehabilitation program for astronauts, combined with 10,000 steps/day. The rehab program consists of 5 days of mental recovery followed by 40 days of physical rehabilitation, including cardio (assumed as running and walking), agility training (assumed as jumping), and strength training (assumed as squats) [62, 63]. Evaluating vertebral loading during these activities requires experimental data, but in the absence of such data, they are estimated based on available data in the literature. Ground Reaction Forces (GRFs) during walking and running at 8 mph are 1.18 BW and 1.76±0.25BW, respectively [60]. Also, the load on the vertebra during walking is 1.0BW. Assuming that the ratio of GRFs during walking and running reflects the ratio of vertebral loads during the same activities, we get

(40) $$\text{Load}\;\mathrm{on}\;\text{vertebra during running}=\frac{1.0}{1.18}\times 1.76\;\mathrm{BW}=1.49\;\mathrm{BW}.$$

Considering the load‐sharing between trabecular and cortical bones in a 55%–45% ratio, the load on vertebral trabecular and cortical bones during running (at 8 mph) is estimated to be 0.82 BW and 0.67 BW, respectively. Similarly, for jumping, with a peak GRF of 3.5BW [69], and assuming the same ratio of GRFs during walking and jumping applies to vertebral loads, we calculate

(41) $$\text{Load}\;\mathrm{on}\;\text{vertebra during jumping}=\frac{1.0}{1.18}\times 3.5\;\mathrm{BW}=2.97\;\mathrm{BW}.$$

Considering the same 55%–45% load‐sharing ratio, the load during jumping on vertebral trabecular and cortical bones is 1.634 BW and 1.337 BW, respectively.

Next, we estimate the load on the vertebra during squats. For a 204 lb. person, vertebral forces during squats range from 1000to2200N [70]. Using the average value of 1600 N, the load for a 165 lb. person is calculated as $1600\times \frac{165}{204}=1294\;N$. Considering the 55% to 45% load‐sharing between trabecular and cortical bones, the corresponding loads are 711.7 N and 582.3 N, respectively. To convert these forces into compressive stresses in MPa, they are divided by the vertebral cross‐sectional area of 1126 ${\mathrm{mm}}^{2}$.

**FIGURE 8:**
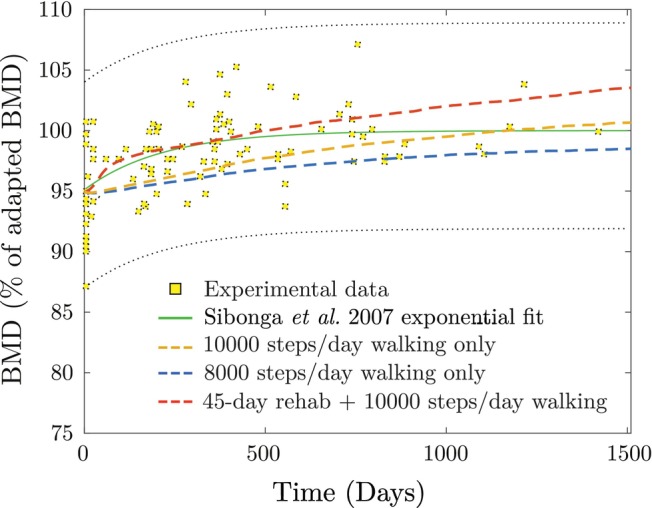
Vertebral bone mass recovery after return to Earth, comparing the predicted change in apparent BMD with real‐life data and the exponential fit from Sibonga et al. for three activity levels: 8000 steps/day, 10,000 steps/day, and a 45‐day rehabilitation program with strength training (running, jumping, and squatting) followed by 10,000 steps/day walking.

Figure 8 shows the simulated vertebral BMD recovery after return to Earth, compared with real‐life astronaut data and the exponential fit from Sibonga et al. [71]. The comparison shows a satisfactory match between the simulated and experimental data across all three loading scenarios, indicating the model's ability to estimate realistic BMD changes.

#### 4.1.2 BMD Loss and Recovery in Bed Rest Studies

We next investigate bone remodeling during prolonged bed rest leading to bone loss from disuse—a common situation for those recovering from major surgery or in a coma. Several studies, including controlled experiments, have been performed on this scenario [59, 64, 72, 73, 74]. In this work, we validate our model against Leblanc et al. [64], which examined bone loss and recovery over 17 weeks of bed rest followed by 6 months of recovery. The study involved six healthy men (19–52 years, 66–80 kg) on strict horizontal bed rest for 17 weeks, only allowed to prop themselves up for eating, reading, or moving to a nearby bathroom a few times per week.

For this validation study, we consider a 75 kg subject and perform validation in three stages, similar to the previous example:

- Stage I: Macroscopic bone adaptation to walking load
- Stage II: BMD loss during bed rest
- Stage III: BMD recovery after bed rest

Stage I involves macroscopic bone adaptation to walking load (8000 steps/day), which is identical to the previous example in Section 4.1.1. Recall that the apparent BMD values at the end of this stage are ${\left(\rho_{\mathrm{app}}\right)}_{\text{trab}}=0.249g/{\mathrm{cm}}^{3}$ and ${\left(\rho_{\mathrm{app}}\right)}_{\text{cort}}=1.31g/{\mathrm{cm}}^{3}$. Also, the final cell populations are ${\left(x_{k}\right)}_{\text{trab}}=11300{\mathrm{mm}}^{-3}$, ${\left(x_{b}\right)}_{\text{trab}}=423{\mathrm{mm}}^{-3}$, and ${\left(x_{c}\right)}_{\text{trab}}=71{\mathrm{mm}}^{-3}$ for trabecular bone and ${\left(x_{k}\right)}_{\text{cort}}=7589{\mathrm{mm}}^{-3}$, ${\left(x_{b}\right)}_{\text{cort}}=272{\mathrm{mm}}^{-3}$, and ${\left(x_{c}\right)}_{\text{cort}}=19{\mathrm{mm}}^{-3}$ for cortical bone.

For Stage II, we estimate the loads acting on the trabecular and cortical regions of the vertebral body during bed rest. It is reported that the intradiscal pressure during bed rest is 20% of the pressure experienced while standing [75]. Therefore, we assume the compressive load on the vertebra during bed rest is 20% of the standing load. The compressive force on the L3‐L4 vertebra during standing is estimated as 0.75 BW [49], meaning the bed rest load is approximately 0.15BW. With trabecular and cortical bones sharing the load in a 55%–45% ratio, the compressive forces are 0.0825BW for trabecular bone and 0.0675BW for cortical bone. Unlike the previous example, no load variability is considered here, and the same load value is applied for each cycle.

For simplicity, we assume that the only load acting on the vertebra during bed rest is from lying down, as the magnitude and duration of loads from other activities (such as raising oneself on an elbow for eating) are negligible. To estimate the number of loading cycles, we approximate it using the Periodic Leg Movements (PLMs) during bed rest, with an average of 28.9 movements per hour [76]. Over 1 day, this equates to approximately 694 loading cycles/day.

**FIGURE 9:**
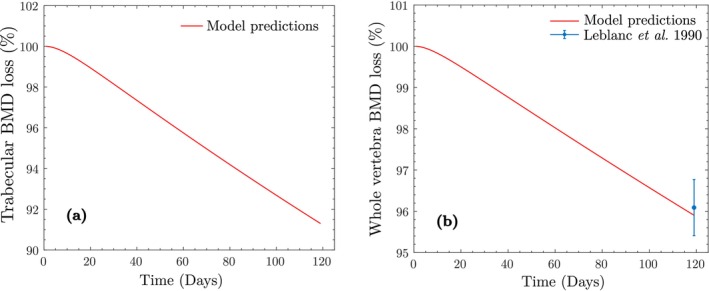
BMD loss during bed rest, showing the change in apparent BMD as a percentage of the adapted BMD predicted using the proposed model (same as shown in Figure 6), due to a reduced activity level during bed rest: (a) Trabecular bone; (b) whole vertebra.

Figure 9 shows the simulated BMD loss in trabecular bone and the whole vertebra after 17 weeks of bed rest (end of Stage II). For the whole vertebra BMD (Figure 9b), the model underpredicts the Leblanc et al. measurement by 0.2% (SD = ±0.7%), which lies within the reported standard deviations, indicating strong agreement with experimental observations and supporting the validity of the model under bed rest conditions. As in the previous example, to estimate the BMD loss for the whole vertebra, we calculate the volume fractions of trabecular $\left(v_{\text{trab}}\right)$ and cortical $\left(v_{\text{cort}}\right)$ bones, and compute the BMD loss as a weighted average based on these fractions. The final cell populations at the end of bed rest are ${\left(x_{k}\right)}_{\text{trab}}=11139.7{\mathrm{mm}}^{-3}$, ${\left(x_{b}\right)}_{\text{trab}}=47{\mathrm{mm}}^{-3}$, and ${\left(x_{c}\right)}_{\text{trab}}=804{\mathrm{mm}}^{-3}$ for trabecular bone, and ${\left(x_{k}\right)}_{\text{cort}}=7033{\mathrm{mm}}^{-3}$, ${\left(x_{b}\right)}_{\text{cort}}=36{\mathrm{mm}}^{-3}$, and ${\left(x_{c}\right)}_{\text{cort}}=252{\mathrm{mm}}^{-3}$ for cortical bone.

**FIGURE 10:**
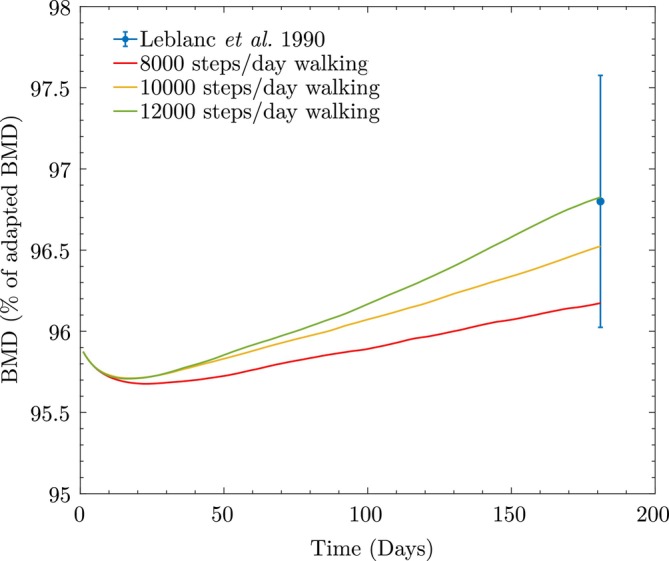
Vertebral bone mass recovery after rehabilitation, comparing the change in apparent BMD as a percentage of the adapted BMD predicted using the proposed model obtained at assumed activity levels, with Leblanc et al.

Moving to Stage III, we simulate BMD recovery after the bed rest period. In the study by Leblanc et al. [64], a controlled exercise program began for four subjects around week 3–4 of the ambulation phase, consisting of supervised 30‐min sessions, 3 times/week for 8 weeks. Afterward, subjects were released and monitored for BMD changes approximately once a month for 6 months. Since details on the type of exercises and walking steps/day during ambulation are unavailable, we assume walking as the primary activity, testing three different activity levels: 8000, 10,000, and 12,000 steps/day. As shown in Figure 10, the predicted BMD values at the end of 6 months lie within or near the experimental range reported by Leblanc et al. (mean = 96.8%, SD = ±0.8%) [64]. The model underpredicts the experimental mean by only 0.6% for the 8000 steps/day case, 0.3% for the 10,000 steps/day case, and shows negligible deviation for the 12,000 steps/day case. All predicted values fall within the reported standard deviation, demonstrating that the model provides physiologically realistic predictions for BMD recovery under varying daily walking activity.

#### 4.1.3 Controlled Study: Yamazaki et al.

Next, we validate our model using the experimental study by Yamazaki et al. [52], which investigated the effect of walking exercise on BMD changes in postmenopausal women with osteopenia/osteoporosis. In this study, 50 postmenopausal women (aged 49–75 years) were recruited, with 32 women participating in an exercise program (exercise group) and 18 serving as the control group. We validate our model against the exercise group using the data in Table 2 extracted from [52], which provides the necessary details such as walking steps/day and the BMD changes over time.

**TABLE 2:** Experimental data from Yamazaki et al. [52] for the exercise group showing the daily walking step count, lumbar spine BMD, and the percentage change of BMD from baseline.

	Baseline	Month 6	Month 12
Daily step count	4256 ± 348	8053 ± 352	8185 ± 315
Lumbar BMD (g/cm^2^)	0.699 ± 0.08	0.703 ± 0.09	0.714 ± 0.09
Percentage change of BMD from baseline	NA	0.47 ± 0.21	1.71 ± 0.85
Frequency of exercise (days/week)	NA	4.2	4.2

**FIGURE 11:**
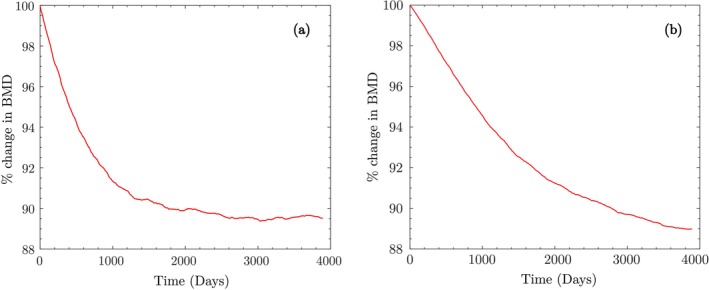
Change in apparent BMD as a percentage of initial BMD during the adaptation of macroscale bone to 4256 steps/day of walking: (a) Trabecular bone and (b) cortical bone.

Firstly, as in the previous validation studies, we assume a subject with initial apparent densities for the trabecular and cortical compartments of the vertebra set to ${\left(\rho_{\mathrm{app}}\right)}_{\text{trab}}=0.246g/{\mathrm{cm}}^{3}$ and ${\left(\rho_{\mathrm{app}}\right)}_{\text{cort}}=1.337g/{\mathrm{cm}}^{3}$, respectively. We then adapt both bone types to the baseline walking load of 4256 steps/day. The percentage change in BMD for trabecular and cortical bone during this adaptation is shown in Figure 11.

For the exercise group, as with previous validation studies, the cell populations ($x_{k},x_{b},x_{c}$) and BMD are initialized using the values at the end of the adaptation phase. The BMD values for trabecular and cortical bones at the end of adaptation are ${\left(\rho_{\mathrm{app}}\right)}_{\text{trab}}=0.22g/{\mathrm{cm}}^{3}$ and ${\left(\rho_{\mathrm{app}}\right)}_{\text{cort}}=1.19g/{\mathrm{cm}}^{3}$, respectively. The corresponding cell populations are ${\left(x_{k}\right)}_{\text{trab}}=8445.5{\mathrm{mm}}^{-3}$, ${\left(x_{b}\right)}_{\text{trab}}=311.5{\mathrm{mm}}^{-3}$, and ${\left(x_{c}\right)}_{\text{trab}}=53.7{\mathrm{mm}}^{-3}$ for trabecular bone, and ${\left(x_{k}\right)}_{\text{cort}}=7026{\mathrm{mm}}^{-3}$, ${\left(x_{b}\right)}_{\text{cort}}=232.5{\mathrm{mm}}^{-3}$, and ${\left(x_{c}\right)}_{\text{cort}}=18.2{\mathrm{mm}}^{-3}$ for cortical bone.

In Stage II, we simulate BMD changes over the first 6 months and compare the percentage change in BMD with the values reported in Table 2. Given that the exercise frequency is 4.2 days/week, walking steps/day for 6 months are set to 8053 for 108 days, while for the remaining 72 days, the walking steps/day are assumed to return to the baseline of 4256 steps/day. Ideally, this would be modeled weekly, with 8053 steps/day for 4.2 days and 4256 steps/day for the remaining 2.8 days. However, for simplicity, we simulate the first 108 days with 8053 steps/day, followed by 72 days with 4256 steps/day. Under the specified assumptions, the simulated percentage change in apparent BMD at the end of 6 months is 0.5%, compared to the experimental value of 0.47%±0.21% reported by Yamazaki et al. [52] (see Table 2), resulting in a deviation of only +0.03%.

**FIGURE 12:**
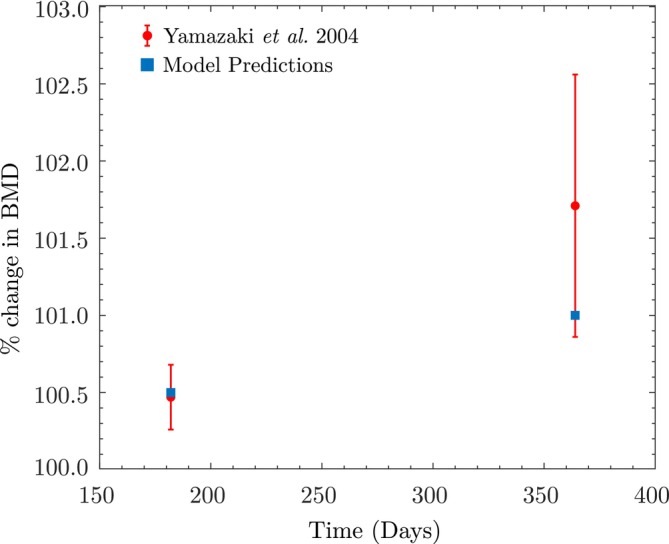
Vertebral bone mass changes simulated using the current model, and compared with the data reported in Yamazaki et al.

For Stage III, we initialize the cell population values $\left(x_{k},x_{b},x_{c}\right)$ and BMD with the values obtained at the end of Stage II. We then simulate the first 108 days with 8185 walking steps/day, followed by 72 days at 4256 steps/day. The simulated percentage change in apparent BMD at the end of 12 months is +1.0%, compared to an experimental range of 1.71%±0.85%, corresponding to a deviation of −0.71% but still within the reported standard deviation, as shown in Figure 12.

Note that Yamazaki et al. conducted their experiments on subjects with osteopenia/osteoporosis. In our model, we do not currently distinguish between healthy and osteoporotic bone, focusing instead on obtaining reasonable estimates for BMD changes. Differentiating between healthy and osteoporotic bone would require further experimental data to parameterize the model specifically for each case. Despite these limitations, our model provides reasonable predictions for BMD changes, demonstrating its potential as a candidate for patient‐specific bone remodeling in the future.

#### 4.1.4 Controlled Study: Iwamoto et al.

Finally, we consider the experimental study by Iwamoto et al. [65], which examined the effects of exercise training and detraining on BMD in postmenopausal women with osteoporosis. In this study, 35 postmenopausal women aged 53–77 years were randomly assigned to three groups: a control group (20 subjects), a 2‐year exercise training group (8 subjects), and a group with 1 year of exercise training followed by 1 year of detraining (7 subjects).

The exercise training consisted of daily brisk walking and gymnastic exercises, including 15 repetitions of straight leg raises, squats, and abdominal/back muscle strengthening. Since specific details about the abdominal and back exercises were unavailable, we focused on validating the control group data, where subjects only performed daily walking. The walking steps per day and corresponding BMD changes for the control group reported in [65] are summarized in Table 3.

**TABLE 3:** Experimental data from Iwamoto et al. [65] for the control group showing the daily walking step count, lumbar spine BMD, and the percentage change of BMD from baseline.

	Baseline	1 Year	2 Years
Daily step count	5280 ± 1432	5028 ± 1008	5384 ± 1248
Lumbar BMD (g/cm^2^)	0.611 ± 0.045	0.617 ± 0.043	0.616 ± 0.044
Percentage change of BMD from baseline	NA	1.01 ± 3.16	0.96 ± 3.39

**FIGURE 13:**
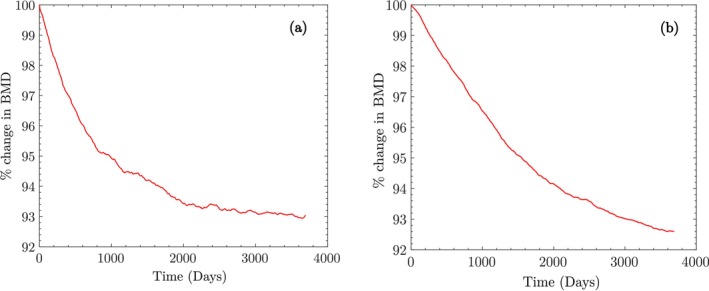
Change in apparent BMD as a percentage of initial BMD during the adaptation of macroscale bone to 5280 steps/day of walking: (a) Trabecular bone (b) Cortical bone.

Similar to the previous validation studies, we assume a subject with initial apparent densities for the trabecular and cortical compartments of the vertebra as ${\left(\rho_{\mathrm{app}}\right)}_{\text{trab}}=0.246g/{\mathrm{cm}}^{3}$ and ${\left(\rho_{\mathrm{app}}\right)}_{\text{cort}}=1.337g/{\mathrm{cm}}^{3}$. We then adapt both bone types to the baseline walking load of 5280 steps/day. The percentage change in BMD for cortical and trabecular bone during this adaptation is shown in Figure 13.

**FIGURE 14:**
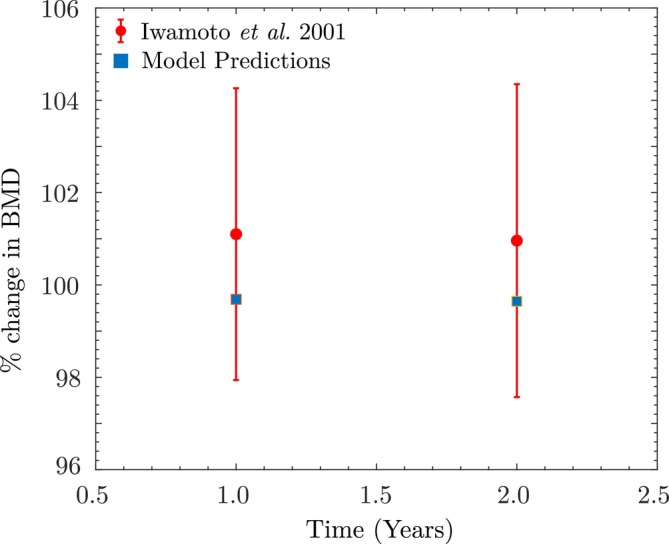
Vertebral bone mass changes simulated using the current model, and compared with the data reported in Iwamoto et al.

Next, we simulate the BMD changes after the first year (Stage II). The cell population and BMD values at the end of the adaptation phase are identical to those used in the previous example in Section 4.1.3. Using 5028 walking steps/day, the resulting BMD change over 1 year is −0.28% (Figure 14), which corresponds to a deviation of approximately −1.29% from the experimental mean of 1.01%±3.16% reported by Iwamoto et al. (also see Table 3).

For Stage III, we simulate the BMD changes over the second year by initializing the cell population values $\left(x_{k},x_{b},x_{c}\right)$ and BMD with the values at the end of Stage II. Using 5384 walking steps/day, we simulate a percentage change in BMD of −0.299% (Figure 14), resulting in a deviation of −1.30% from the reported value of 0.96%±3.39% in [65]. In both cases, the model predictions fall within the reported standard deviations, indicating good agreement with the observed long‐term BMD trends under daily walking activity.

### 4.2 Shape Optimization to Capture Microscale Variations

After validating our macroscale model with multiple datasets, we move forward with shape optimization in ABAQUS, using a volume constraint as input. As detailed in Section 3.2, we begin by estimating the changes in BMD, $\Delta \rho_{\mathrm{app}}$, due to walking, and then calculate the volume constraint f. This constraint is applied during the shape optimization process. Additionally, we minimize the maximum von Mises stress, as outlined in Section 3.2, to ensure a more uniform stress distribution throughout the domain.

**FIGURE 15:**
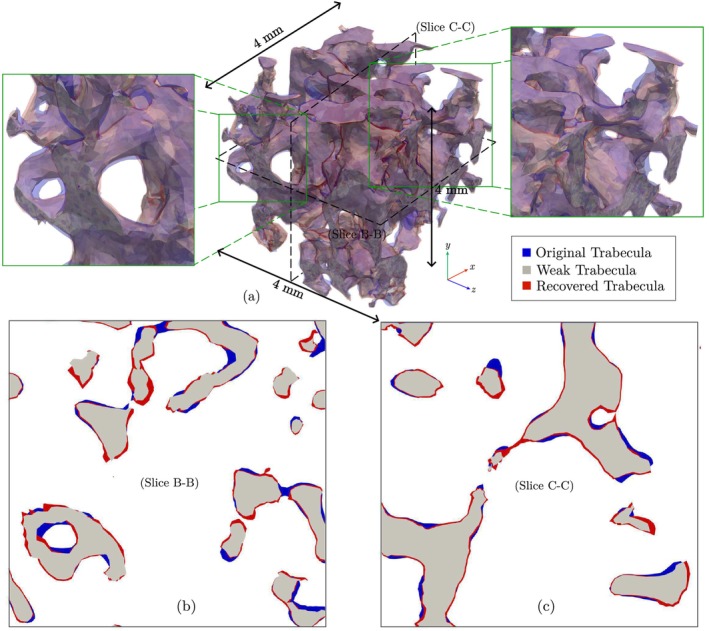
Shape optimization results showing the comparison between the initial trabecula, the weakened trabecula due to space travel, and the recovered trabecula after 1500 days.

In this section, this process is utilized to estimate surface changes resulting from bone loss due to space travel, as well as subsequent recovery (see Section 4.1.1). The initial model focuses on trabecular bone, labeled as “Original Trabecula” in Figure 15, with a tissue density of ${\left(\rho_{t}\right)}_{\text{trab}}=1.368g/{\mathrm{cm}}^{3}$ and an initial porosity of $n_{0}=82\%$. This corresponds to an initial bone volume fraction of ${\left(\frac{\mathrm{BV}}{\mathrm{TV}}\right)}_{0}=18\%$, and an apparent density of ${\left({\left(\rho_{\mathrm{app}}\right)}_{\text{trab}}\right)}_{I}=0.2485g/{\mathrm{cm}}^{3}$. At the end of Stage I (the adaptation phase), the results in Figure 6 show an approximate 1% increase in the apparent density of trabecular bone, yielding a new apparent density of $\left(\left(\rho_{\mathrm{app}}\right)\text{trab}\right)I=0.2485g/{\mathrm{cm}}^{3}$. Assuming a constant tissue density, the porosity at the end of Stage I is estimated as

(42) $$n_{I}=1-\frac{{\left({\left(\rho_{\mathrm{app}}\right)}_{\text{trab}}\right)}_{I}}{{\left(\rho_{t}\right)}_{\text{trab}}}=1-\frac{0.2485}{1.368}=81.83\%.$$

From this, the volume fraction can be calculated as ${\left(\frac{\mathrm{BV}}{\mathrm{TV}}\right)}_{I}=18.17\%$. This provides a volume constraint for the shape optimization process $f_{0‐I}=1.0094$, which is the ratio of ${\left(\frac{\mathrm{BV}}{\mathrm{TV}}\right)}_{I}$ and ${\left(\frac{\mathrm{BV}}{\mathrm{TV}}\right)}_{0}$.

Moving to Stage II, the apparent density of trabecular bone at the end of this stage (after space travel) has decreased by approximately 9.8% (Figure 7), resulting in an apparent density of ${\left({\left(\rho_{\mathrm{app}}\right)}_{\text{trab}}\right)}_{\mathrm{II}}=0.2242g/{\mathrm{cm}}^{3}$. This bone, labeled as “weak trabecula” in Figure 15 and colored in “gray,” appears visibly thinner. The corresponding porosity, $n_{\mathrm{II}}$, is then estimated using the same approach as before to be 83.61%. This yields a volume fraction of ${\left(\frac{\mathrm{BV}}{\mathrm{TV}}\right)}_{\mathrm{II}}=16.39\%$ and thereby a volume constraint of $f_{I‐\mathrm{II}}=0.9024$ for the shape optimization process at the end of Stage II.

For Stage III (bone mass recovery 1500 days after space travel), we assume 10,000 walking steps/day, as shown by the yellow dotted line in Figure 8. After recovery, the trabecular bone has 2.23% higher apparent BMD than the adapted bone, giving ${\left({\left(\rho_{\mathrm{app}}\right)}_{\text{trab}}\right)}_{\mathrm{III}}=0.2540g/{\mathrm{cm}}^{3}$. The recovered trabecular bone, colored “red” in Figure 15, is visibly thicker than the “weak trabecula”, indicating that recovery has strengthened the bone. The corresponding porosity, volume fraction, and volume constraints at the end of Stage III are then evaluated as $n_{\mathrm{III}}=81.43\%$, ${\left(\frac{\mathrm{BV}}{\mathrm{TV}}\right)}_{\mathrm{III}}=18.57\%$, and $f_{\mathrm{II}‐\mathrm{III}}=1.133$, respectively. As shown in Figure 15, this shape optimization strategy can capture volumetric changes of the bone at the microscale, allowing for a detailed analysis of bone structure adaptations and recovery at various stages.

It is worth noting that, for the sample shown in Figure 15 consisting of approximately 0.5 million elements, each of the shape optimization simulations, that is, Stage I, II, and III, required approximately 12 h when executed on a compute node with 2.4 GHz Intel Xeon E5‐2680 v4 processors (28 cores per node).

#### 4.2.1 Sensitivity Analysis

Next, the study by Yamazaki et al. [52] is selected to conduct a thorough sensitivity analysis, exploring various possible values for key parameters such as m, $\beta_{\text{k}}$, trabecular bone reference stimulus ${\left(S_{0}\right)}_{\text{trab}}$, and cortical bone reference stimulus ${\left(S_{0}\right)}_{\text{cort}}$. This sensitivity analysis is crucial because specific values for these parameters were assumed, given the challenge of accurately estimating them without extensive experimentation.

**FIGURE 16:**
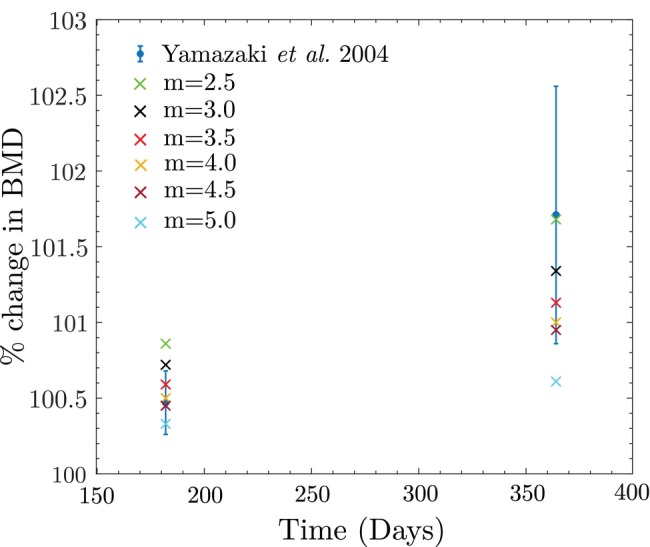
Sensitivity of the model to the value of m studied using Yamazaki et al. data.

First, the sensitivity of the model to the parameter m is examined. In Section 3.1.5, we assumed m=4 based on studies by Whalen et al. [55] and Beaupre et al. [6]. Notably, Whalen et al. [55] proposed a range of 2.3–4.8 for a similar empirical exponent related to daily applied loading history. Based on this, several values of m, ranging from 2.5 to 5.0 with increments of 0.5, are tested. The corresponding results are presented in Figure 16, showing that an increase in m leads to a decrease in the percentage change in BMD. It is observed that values of m in the range of 3.5 and 4.5 provide BMD predictions within the standard deviation reported by Yamazaki et al. [52] at both 6 months and 1 year. In contrast, m=5.0 gives predictions within the standard deviation only at 6 months, while m=2.5 and m=3.0 yield predictions within the standard deviation only at 1 year. This does not imply that m=2.5, 3.0, and 5.0 are essentially poor fits, as these outliers may represent subjects with specific health conditions resulting in atypical bone remodeling rates [77]. Additionally, it is important to note that the subjects in Yamazaki et al. [52] were diagnosed with osteopenia or osteoporosis. Nevertheless, values of m between 3.5 and 4.5 provide the best fit for the data from Yamazaki et al. [52], although its exact value varies between individuals and requires tuning for each subject.

Next, we analyze the sensitivity of the model to the values of $\beta_{k}$. As mentioned in Section 3.1.4, osteocytes exhibit a much longer lifespan compared to osteoclasts and osteoblasts, with an average lifespan of 25 years, and some living up to 50 years [42]. Assuming an exponential decay in cell population, the half‐life of osteocytes is initially set to 10 years, resulting in $\beta_{k}=1.9e-04$. This assumption is tested by using multiple values for $\beta_{k}$, with half‐lives ranging from 7.5 to 15 years, in increments of 2.5 years. For a half‐life of 7.5 years, $\beta_{k}=2.5e-04$, for 12.5 years, $\beta_{k}=1.5e-04$, and for 15 years, $\beta_{k}=1.27e-04$.

**FIGURE 17:**
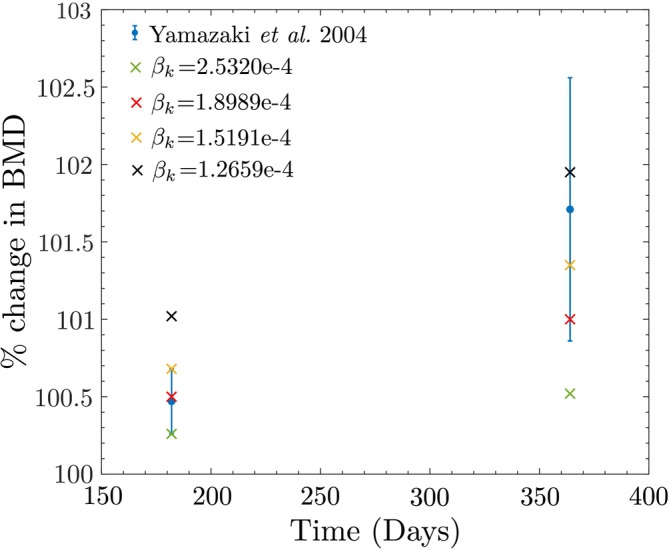
Sensitivity of the model to the value of $\beta_{k}$ studied using Yamazaki et al. data.

The results are shown in Figure 17, where it is observed that for $\beta_{k}=1.5e-04$ and $\beta_{k}=1.9e-04$, the predicted BMD values fall within the standard deviation range reported by Yamazaki et al. [52] at both 6 months and 1 year. However, $\beta_{k}=2.5e-04$ underpredicts BMD at 1 year, while $\beta_{k}=1.27e-04$ overpredicts BMD at 6 months. This suggests that assuming an average osteocyte half‐life of 10–12.5 years provides the best fit for the data reported by Yamazaki et al. [52]. However, these results do not exclude other values of $\beta_{k}$, as Yamazaki et al. [52] only included 50 subjects. Therefore, a larger and more diverse group would be required to draw definitive conclusions.

A sensitivity analysis was also conducted on the trabecular and cortical bone reference stimuli ${\left(S_{0}\right)}_{\text{trab}}$ and ${\left(S_{0}\right)}_{\text{cort}}$, which are influenced by the values assumed for the reference apparent density. For trabecular bone, the apparent density typically ranges from $0.05-0.36\frac{g}{{\mathrm{cm}}^{3}}$, with an average vertebral BMD around 0.20 $\frac{g}{{\mathrm{cm}}^{3}}$ [78]. Based on this, we tested 0.175, 0.20, 0.225, 0.25, and 0.275 $\frac{g}{{\mathrm{cm}}^{3}}$ for the trabecular bone reference stimulus, at which no net change in BMD occurs when walking 5000 steps per day. For validation studies, the threshold BMD values for trabecular and cortical bone are assumed to be 0.225 $\frac{g}{{\mathrm{cm}}^{3}}$ and 1.20 $\frac{g}{{\mathrm{cm}}^{3}}$, respectively.

**FIGURE 18:**
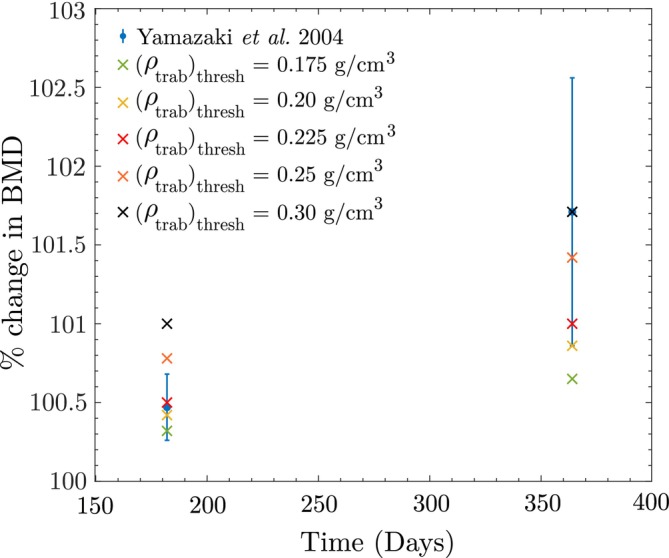
Sensitivity of the model to the value of the trabecular bone reference stimulus $S_{0}$ studied using Yamazaki et al. data.

For each assumed trabecular threshold BMD value, the validation of the study by Yamazaki et al. [52] is repeated, keeping the cortical bone reference fixed at 1.20 $\frac{g}{{\mathrm{cm}}^{3}}$. The corresponding results are illustrated in Figure 18, showing that assuming trabecular bone thresholds corresponding to BMDs of 0.20 and 0.225 $\frac{g}{{\mathrm{cm}}^{3}}$ yield predicted BMD values at both 6 months and 1 year within the standard deviation reported by Yamazaki et al. [52]. However, for BMD thresholds of 0.25 and 0.275 $\frac{g}{{\mathrm{cm}}^{3}}$, there is an overestimation at 6 months, while a threshold of 0.175 $\frac{g}{{\mathrm{cm}}^{3}}$ underestimates at 1 year. The exact value for this parameter varies between individuals, and accurate estimation would require further studies.

**FIGURE 19:**
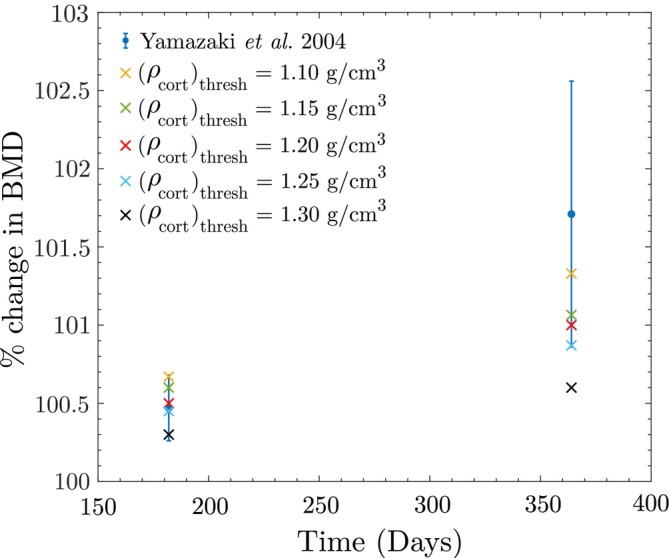
Sensitivity of the model to the value of the cortical bone reference stimulus $S_{0}$ studied using Yamazaki et al. data.

Similarly, to assess the sensitivity of the model to the cortical bone reference stimulus ${\left(S_{0}\right)}_{\text{cort}}$, several values for bone reference BMD are studied: 1.10, 1.15, 1.20, 1.25, and 1.30 $\frac{g}{{\mathrm{cm}}^{3}}$. These values are chosen based on data from Osterhoff et al. [67], which indicates that cortical bone BMD typically ranges between 1.00 and 1.35 $\frac{g}{{\mathrm{cm}}^{3}}$. For each assumed value, the validation of the study by Yamazaki et al. [52] is repeated, keeping the trabecular bone reference fixed at 0.225 $\frac{g}{{\mathrm{cm}}^{3}}$. The corresponding results are shown in Figure 19, where for the assumed cortical threshold BMD values of 1.10−1.25 $\frac{g}{{\mathrm{cm}}^{3}}$ the predicted BMD values at both 6 months and 1 year fall within the range reported in [52]. Note that 1.30 $\frac{g}{{\mathrm{cm}}^{3}}$ is near the upper limit of typical cortical bone BMD values, it is reasonable to suggest that it might be too high a threshold for the general population. However, it could be valid for individuals with bone remodeling disorders that impair the ability to increase bone BMD.

The sensitivity analysis presented in this section demonstrates that the proposed model can provide reasonable estimates for the percentage change in BMD across various assumed values for key parameters, including m, $\beta_{k}$, and the trabecular and cortical bone reference stimuli ${\left(S_{0}\right)}_{\text{trab}}$ and ${\left(S_{0}\right)}_{\text{cort}}$. While determining the exact values for these parameters is challenging and would require extensive experimental studies, the validation and sensitivity analyses conducted here show that the model effectively captures the variability of the reported experimental data. This positions it as a strong candidate for future studies on patient‐specific bone remodeling.

### 4.3 Effect of Bone Remodeling on Vertebral Fracture

In this section, we investigate the impact of bone remodeling in the trabecular microstructure on vertebral strength. To create high‐fidelity FE models for predicting the bone remodeling process, including the density and shape changes of the microstructure, we employ the integrated modeling framework (named ReconGAN) introduced in reference [66] to construct a realistic virtual geometric model of the vertebral body. This process involves three main steps: (i) recreating the trabecular bone microstructure using a 3D deep convolutional generative adversarial network (DCGAN), forming the initial trabecula; (ii) applying shape optimization to estimate volumetric changes in the trabecular microstructure; (iii) extracting the vertebra's cortical shell from digital CT images of the patient's spine and integrating it with the trabecular microstructure.

**FIGURE 20:**
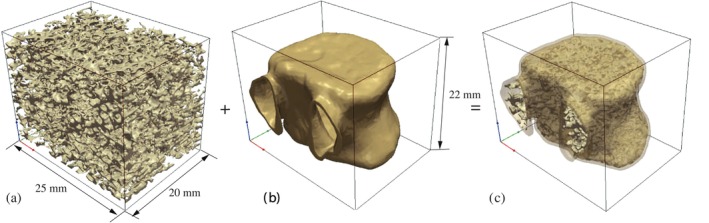
Creating the vertebra geometrical model by engraving the synthesized trabecular microstructure and integrating it with the cortical shell extracted from patient CT data.

The process of creating a geometrical model of the entire vertebra is illustrated in Figure 20. The cuboidal trabecular microstructure, generated by ReconGAN, is combined with the cortical shell extracted from the CT scan. This engraving and embedding process is implemented using Python code. However, an abrupt transition between the trabecular region and the cortical shell can create sharp angles at the interface. To address this, shape optimization is applied to smooth out the trabecular‐to‐cortical transition to minimize stress concentrations between these regions and ensure a more uniform structure in the resulting vertebra model.

The entire vertebra model, generated as described, is converted into a high‐fidelity finite element (FE) model using the commercial software Simpleware, consisting of 7 million elements. In the FE model of the spinal vertebral body, it is crucial to account for the heterogeneous material properties of the trabecular bone. This is done by mapping the elastic modulus, calculated from grayscale (GS) values in the voxel‐based microstructure, onto the mesh elements. These mesh elements may not necessarily align with the original image voxels after segmentation. Additionally, sections of the intervertebral disc (IVD) are included in the FE model, with effective material properties assigned: an elastic modulus $E_{\mathrm{IVD}}=17$ MPa and a Poisson's ratio $\nu_{\mathrm{IVD}}=0.4586$. These IVD sections are modeled as linear elastic materials, improving the accuracy of simulating the in vivo loads applied to the vertebra. The boundary conditions involve constraining the displacement degrees of freedom on the bottom surface of the lower IVD. A uniform downward displacement is applied at a rate of ${\dot{u}}_{z}=0.05$ mm/s to the upper IVD, simulating realistic loading conditions on the vertebra. This setup enhances the realism of load‐bearing simulations in the model.

Furthermore, the cuboidal trabecular microstructure from Figure 20 is used as the initial trabecular microstructure to simulate volumetric changes through the shape optimization procedure proposed in this study. The initial volume fraction of this microstructure is around 82%, aligning with the initial volume fraction of the smaller trabecular microstructure in Figure 15. This allows the same percentage volume changes from the space travel example in Section 4.2 to be applied, specifically a volume constraint of 1.0094 for Stage I, 0.9024 for Stage II, and 1.133 for Stage III. This assumption is valid because uniform compression is the only load type considered, and both the initial microstructure from Figure 20 and the smaller trabecular sample from Figure 15 are cuboidal. Consequently, all elements experience identical uniform stresses. The corresponding shape optimization simulations are then performed using ABAQUS, and the modified trabecular microstructures are obtained after each stage. It is worth noting that each of the shape optimization simulations required approximately 8 days of runtime on a compute node equipped with dual 2.4 GHz Intel Xeon E5‐2680 v4 processors (56 cores total). Subsequently, three FE models of the vertebra, corresponding to the adapted state (Stage I), weakened vertebra (Stage II), and recovered vertebra (Stage III), are generated by integrating the trabecular microstructure with the cortical bone.

Following this, the vertebral compression fracture (VCF) simulations were conducted in ABAQUS using a continuum damage model described in [66], which estimates the onset and progression of damage within the bone tissue. To avoid convergence issues caused by fully damaged elements (which have zero stiffness), an element deletion strategy was employed. This strategy removes these elements, preventing severe distortion during the simulation. The simulations were executed on an Intel Xeon Platinum 8468H processor with 48 cores. Due to the substantial element count within the FE models, parallel computing was utilized, leveraging all 48 available cores. The simulation runtime was approximately 25 h. Additionally, a self‐contact model was used to prevent fractured bone segments from overlapping or penetrating each other under compressive forces. This self‐contact model was crucial for maintaining the realism of the simulation, ensuring that the fractured bone behaved correctly, and contributing to the overall accuracy of the results.

**FIGURE 21:**
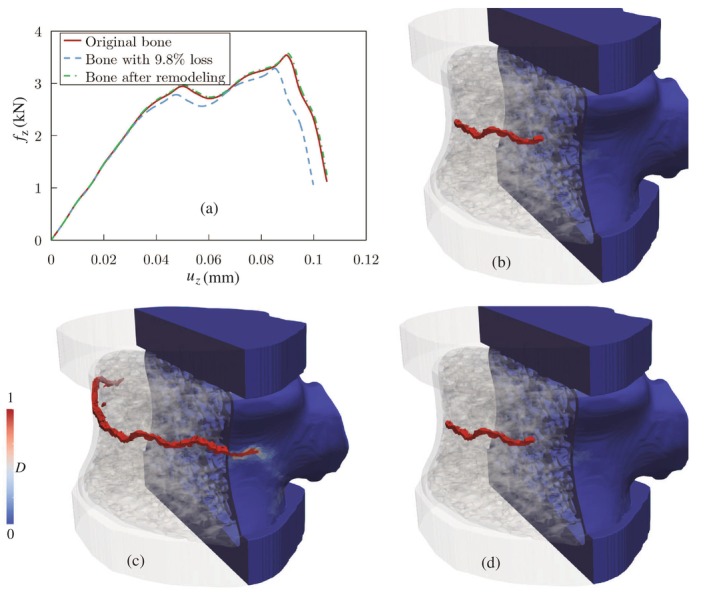
(a) Force–displacement responses of the vertebra during three stages of the remodeling process subjected to uniform compression; (b–d) damage patterns in these models under compression ($u_{z}=0.9$ mm). Note that $u_{z}$ is the applied displacement boundary condition in the z direction, while $f_{z}$ refers to the resultant reaction force along the top surface of the vertebra.

The mechanical behavior of the vertebral model during the entire remodeling process, including the fracture strength and toughness of the vertebra, is analyzed in this study. Figure 21 presents a detailed graphical representation of the vertebra's mechanical behavior during bone remodeling. This complex physiological process results in a series of changes in the trabecular bone, which is accurately captured in the model. Initially, during the remodeling phase, there is a significant decrease in bone density, specifically a 9.8% reduction. Following this, the bone density recovers, nearly returning to its pre‐remodeling levels. The effects of this density fluctuation are clearly shown in Figure 21.

The clinical impact of vertebral trabecular bone density changes during the three stages of the remodeling process is summarized in Table 4. The trabecular bone experiencing a 9.8% density loss exhibited a reduction in both peak load (from 3.532 to 3.28 kN) and energy absorption capacity (from 0.243 to 0.218 J), indicating compromised mechanical performance. Following bone remodeling, mechanical properties were largely recovered, as evidenced by restored peak load (3.534 kN) and energy absorption (0.245 J), closely matching those of the original trabecular bone. This recovery underscores the significance of the remodeling process in maintaining vertebral structural integrity and resistance to mechanical stress.

**TABLE 4:** Clinical impact of density changes of the vertebra during three stages of the remodeling process.

	Peak load (kN)	Energy absorption (J)
Original trabecular bone	3.532	0.243
Trabecular bone with 9.8% density loss	3.28	0.218
Trabecular bone after remodeling	3.534	0.245

One key observation is the reduced strength of the entire vertebra due to the bone density loss, quantified as a 7% decrease in vertebral strength, highlighting the critical role that density plays in the structural integrity of the bone. Additionally, the figure reveals an important insight into the onset of damage during the remodeling process. The damage pattern presented in Figure 21b,c provides a compelling comparison of damage onset in different bone conditions. When the displacement parameter $\left(u_{z}\right)$ reaches 0.9 mm, a notable initiation of damage is observed in the original bone. In contrast, in the bone that has experienced density reduction, not only has the damage begun at the same displacement level, but it has also propagated significantly. This accelerated progression of damage in the lower‐density bone underscores the crucial role of bone density in structural resilience and its increased susceptibility to damage under mechanical stress. The differences observed at this displacement threshold provide valuable insights into the mechanical behavior of bones under varying conditions of density, illustrating the vulnerability of bones with reduced density compared to their original counterparts. Furthermore, the results align with clinical findings, which consistently report that VCFs predominantly occur in the anterior region of the vertebrae [79, 80, 81]. This alignment reinforces the relevance and validity of the findings presented in this study.

Additionally, the force‐displacement curves for the bone post‐remodeling closely resemble those of the original bone, highlighting a significant similarity. This is clearly illustrated in Figure 21b,d, where the damage patterns in both the original and post‐remodeled bone exhibit remarkable parallels. This resemblance suggests that, despite the temporary phase of density loss and subsequent recovery, the mechanical behavior of the bone after remodeling aligns closely with its pre‐remodeling state. Such a finding is crucial as it offers valuable insights into the bone's structural resilience and its capacity to recover mechanical properties after undergoing remodeling. This observation has important implications for bone physiology and medical treatments, especially concerning strategies for maintaining bone health and optimizing rehabilitation after conditions like osteoporosis or injury‐induced remodeling. Understanding the bone's ability to regain its original mechanical behavior post‐remodeling opens up new possibilities for developing effective therapeutic interventions.

## 5 Limitations of the Proposed Model

Despite presenting a comprehensive theoretical and modeling framework, together with a partial validation of the model and sensitivity analysis, the current study has some limitations that warrant further investigation in the future. It is essential to enumerate these limitations, caused by a lack of precise, person‐specific estimates of applied loads, geometrical features, and material properties in our biomechanical models, which affect the accuracy of our simulation results. The purpose of this study was to illustrate overall trends rather than provide exact values.

Firstly, the experimental data used to calibrate model parameters in Sections 3.1.4 and 3.1.5 were derived as reasonable estimates from the literature. Additional comprehensive experimental studies are necessary to obtain precise parameter values tailored to individuals with varying health conditions (e.g., osteoporosis, osteoarthritis, and Paget's disease) and under diverse conditions (e.g., microgravity on the International Space Station). Furthermore, the effects of space radiation on bone cells, as highlighted in prior research [82], were not accounted for in our study. Additionally, the study focuses solely on compressive loading of the vertebrae, which is a simplification. During different physical activities, vertebrae are subjected to anterior/posterior and lateral shear loads in addition to compressive forces. This omission represents a limitation that should be addressed in future work to provide a more comprehensive understanding of vertebral biomechanics. Nevertheless, dynamic loading is incorporated into this study, assuming a standard deviation of 7.5%, as described in Section 3.1.5. This dynamic consideration adds depth but does not fully encompass the complexity of multi‐directional loads.

Additionally, the cross‐sectional area of the lumbar spine used in this study (1126 ${\mathrm{mm}}^{2}$) was derived from a study involving postmenopausal female patients [51]. Other studies in the literature have reported that the mean L4 cross‐sectional area is approximately 1330 ${\mathrm{mm}}^{2}$ and 1050 ${\mathrm{mm}}^{2}$, respectively, in middle‐aged men and women who are moderately active [83]. Averaging these values yields 1190 ${\mathrm{mm}}^{2}$, which is in a similar range to the value used in this study. Nevertheless, a more rigorous approach would involve calibrating the model separately for different demographic groups or, more precisely, developing person‐specific models in future works.

In the validation study on astronauts in space (Section 4.1.1), we assumed that the activity level of an astronaut in the International Space Station (ISS) was 16% of the typical activity level on Earth. Since astronauts experience mechanical loading in compression only during certain physical activities, a more thorough approach would consider the periods when astronauts are in a lower or unloaded state. Additionally, intervertebral disc (IVD) swelling may induce tension in the vertebrae during spaceflight, which has not been accounted for in this study. Further experimental data is required to determine accurate loading conditions in such environments.

Lastly, a recent study on mechanical loading of the spine during various physical activities [84] reported loads experienced by the L4/L5 vertebrae during activities such as jumping (4.8−5.2 times body weight) and squats (1700−1900 N, an approximate range extrapolated from a plot). The values used in the present study (Section 4.1.1) were approximations based on previous research and may differ from these recent findings. It is essential to use more accurate loading values tuned to specific activities and individuals to obtain more precise estimates.

## 6 Conclusion

This study presents an FE‐based numerical framework for studying bone remodeling through a mechano‐biological approach, focusing on bone cell dynamics and the evolution of BMD at the macroscale. Using the apparent BMD percentage changes at the macroscale, we estimate the ratio of the final and initial trabecular volumes, which is then used as a volume constraint in a shape optimization algorithm. The shape optimization algorithm, which minimizes the maximum von Mises stress as its objective function, simulates the volumetric changes at the microscale. Several preliminary validation studies were conducted using human vertebra bone data available in the literature, covering both uncontrolled and controlled studies, including bone loss and recovery in NASA astronauts, bed rest, and experiments by Yamazaki et al. [52] and Iwamoto et al. [65]. Simulating these studies demonstrated that the BMD change predictions made by the proposed mechano‐biological model fall within the range of reported measurements. A detailed sensitivity analysis, using Yamazaki et al. [52] as a reference, further demonstrated the model's ability to capture the experimental BMD percentage changes from physical activity using different combinations of input parameters. Additionally, we compared the Vertebral Compression Fracture (VCF) simulation between the weakened vertebral bone due to space travel and the recovered vertebral bone, in which the proposed framework has been used to estimate the volumetric and morphological changes. This qualitative comparison shows that the peak forces in the original and recovered vertebral bones are higher than those in the weakened bone, indicating that the proposed model can simulate bone remodeling changes in a realistic scenario.

Future work will involve large‐scale model validation using patient‐specific data, enabling parameterization of the model for personalized treatment. This is particularly relevant for patients with spinal metastasis undergoing vertebroplasty, where cement is injected to stabilize the spine [32, 66, 85, 86]. Another potential application is in spinal fusion surgery, where pedicle screws connect multiple vertebrae [87]. Given the computational intensity of bone remodeling simulations, deep learning techniques could be employed to predict remodeled trabecular bone microstructures based on initial conditions, providing rapid predictions in clinical settings. Artificial intelligence‐based methods have shown promise in accelerating numerical simulations by learning complex mappings and reducing computational costs [88]. Such methods could facilitate quick estimations of bone volume changes based on activity levels in a very short time. Furthermore, incorporating the effects of radiation and subsequent model parameterization could enhance recovery protocol designs for patients undergoing radiation therapy, making this framework even more valuable in clinical applications.

## Nomenclature

**Symbol Description**
$X_{i}$
: active population of cell type i=b,c in the proposed model; threshold functions i=k,b,c in Rapisarda et al.
$\beta_{\text{k}},\;\beta_{\text{b}},\;\beta_{\text{c}}$
: apoptotic rates of osteocytes, osteoblasts, and osteoclasts
$\rho_{\mathrm{app}}$
: apparent density at the macroscale
$\alpha_{b},\;\alpha_{c}$
: birth rates of osteoblasts and osteoclasts
$\gamma_{\mathrm{bk}},\;\gamma_{c}$
: conversion rate of osteoblasts into osteocytes and differentiation rate of osteoclasts
${\left(\mathrm{BV}⁄\mathrm{TV}\right)}_{\text{i}},\;{\left(\mathrm{BV}⁄\mathrm{TV}\right)}_{\text{f}}$
: initial and final bone volume fraction
$\rho_{0}$
: initial apparent density
ρ
: local bone tissue density (g/cm^3^)
S
: mechanical stimulus
$S^{-}$
: negative stimulus component (resorption)
N
: number of load cycles considered
$x_{\text{b}}$
: osteoblast population density
$x_{\text{c}}$
: osteoclast population density
$x_{\text{k}}$
: osteocyte population density
ν
: Poisson's ratio
n
: porosity, $n=1-\rho_{\mathrm{app}}/\rho_{t}$
$S^{+}$
: positive stimulus component (formation)
$\kappa \left(\phi \right),\;H\left(\phi \right)$
: regularisation and Heaviside functions in cell ODEs
$n_{i}$
: repetitions of load cycle i
m
: stimulus non‐linearity exponent
U
: strain‐energy density
$S_{0}$
: threshold stimulus for equilibrium
$\rho_{\text{t}}$
: tissue density
$S_{\text{total}}$
: total stimulus over multiple load cycles
f
: volume‐fraction ratio, $f={\left(\mathrm{BV}/\mathrm{TV}\right)}_{f}/{\left(\mathrm{BV}/\mathrm{TV}\right)}_{i}$
$\sigma_{\text{vm}}$
: von Mises stress (shape‐optimization objective)
η
: weighting factor for osteocyte influence in S
E
: Young's modulus
BV⁄ TV
: bone volume fraction (bone volume/total volume)

## Ethics Statement

The authors have nothing to report.

## Conflicts of Interest

The authors declare no conflicts of interest.

## Data Availability

The data that support the findings of this study are available from the corresponding author upon reasonable request.
